# Large Underestimation of Intraspecific Trait Variation and Its Improvements

**DOI:** 10.3389/fpls.2020.00053

**Published:** 2020-02-13

**Authors:** Jing Yang, Jiahui Lu, Yue Chen, Enrong Yan, Junhua Hu, Xihua Wang, Guochun Shen

**Affiliations:** ^1^ Tiantong National Station for Forest Ecosystem Research, School of Ecological and Environmental Sciences, East China Normal University, Shanghai, China; ^2^ Shanghai Institute of Pollution Control and Ecological Security, Shanghai, China; ^3^ Chengdu Institute of Biology, Chinese Academy of Sciences, Chengdu, China

**Keywords:** individual variation, trait variability, coefficient of variation, CV estimator, bias, Tiantong

## Abstract

Intraspecific trait variation (ITV) is common feature of natural communities and has gained increasing attention due to its significant ecological effects on community dynamics and ecosystem functioning. However, the estimation of ITV *per se* has yet to receive much attention, despite the need for accurate ITV estimation for trait-based ecological inferences. It remains unclear if, and to what extent, current estimations of ITV are biased. The most common method used to quantify ITV is the coefficient of variation (CV), which is dimensionless and can therefore be compared across traits, species, and studies. Here, we asked which CV estimator and data normalization method are optimal for quantifying ITV, and further identified the minimum sample size required for ±5% accuracy assuming a completely random sample scheme. To these ends, we compared the performance of four existing CV estimators, together with new simple composite estimators, across different data normalizations, and sample sizes using both a simulated and empirical trait datasets from local to regional scales. Our results consistently showed that the most commonly used ITV estimator (*CV*_1_= *σ_sample_*/*μ_sample_*), often underestimated ITV—in some cases by nearly 50%—and that underestimation varies largely among traits and species. The extent of this bias depends on the sample size, skewness and kurtosis of the trait value distribution. The bias in ITV can be substantially reduced by using log-transforming trait data and alternative CV estimators that take into consideration the above dependencies. We find that the *CV_4_* estimator, also known as Bao's CV estimator, combined with log data normalization, exhibits the lowest bias and can reach ±5% accuracy with sample sizes greater than 20 for almost all examined traits and species. These results demonstrated that many previous ITV measurements may be substantially underestimated and, further, that these underestimations are not equal among species and traits even using the same sample size. These problems can be largely solved by log-transforming trait data first and then using the Bao's CV to quantify ITV. Together, our findings facilitate a more accurate understanding of ITV in community structures and dynamics, and may also benefit studies in other research areas that depend on accurate estimation of CV.

## Introduction

Intraspecific trait variation (ITV) is the overall difference in traits values among conspecific individuals in one or more traits, such as height, specific leaf area, and wood density (Albert et al., 2011). Such variation widely exists in nature (Darwin, 1859; Siefert et al., 2015) and has large ecological effects on population dynamics (Agashe, 2009; Abbott and Stachowicz, 2016), community assembly (Siefert and Ritchie, 2016; Griffiths et al., 2018), and ecosystem functioning (Bukowski and Petermann, 2014; Souza et al., 2017). Because of its wide ranging ecological consequences, the ecological effects of ITV and the extent of ITV are increasingly attracting research attention (Des et al., 2018).

Accurate estimation of ITV is essential for fully understanding species distributions and abundances from a trait-based perspective because the absolute extent of ITV is thought to be closely linked with species tolerances to the abiotic environment and responses to neighborhood interactions (Clark, 2010). Specifically, it is hypothesized that, because species' responses to the environment manifest through functional traits, the higher the ITV of a species is, the more diverse abiotic environments the species may be able to adapt to (Umaña et al., 2015). Therefore, if we underestimate ITV, species' distributions across heterogeneous environments might be underestimated as well (Helsen et al., 2017). Consequently, their resilience to current environmental fluctuation may be underestimated and their extinction risk overestimated. Similarly, the magnitude of ITV is thought to be linked with niche overlap among species (Li et al., 2017). Large ITVs may increase species niche overlap and interactions (Williams et al., 2017) and that in turn promote or hamper species coexistence (Hart et al., 2016). If estimated ITVs of co-occurring species were biased, we likely largely bias the estimation of interspecific interactions and lose accuracy in the predictions of species abundance and coexistence status. However, compared to the current enthusiasm surrounding the ecological effects of ITV, little attention has been paid to the estimation of ITV itself (but see Mitchell and Bakker, 2014). At present, it is still unclear whether ITV estimation is unbiased and accurate enough to facilitate reasonable ecological inferences.

Most current studies use the coefficient of variation (CV = σ/μ) to quantify the absolute extent of ITV and evaluating whether ITV varies among species or traits, and ultimately in thinking about the response of populations or communities to environmental change (see our literature review in **Tables 1** and **S1**). As a single value summary statistic, CV is less informative than other quantifications of ITV (e.g., parametric probability distribution), but it is simple and its definition does not require *ad hoc* assumptions of the underlying probability distributions for each trait. Importantly, CV is unitless, and thus offers a convenient way to directly compare variation (e.g., trait-based niche width) among species with different abundances under various environments (Helsen et al., 2017). For example, comparative studies have shown a positive relationship between species ITV and niche breadth (Clark, 2010): species with larger ITV tend to have larger geographical ranges than species with smaller ITV (Brown, 1984).

**Table 1:** A summary of CV estimation methods, data normalizations and the minimum number of samples for each species used in conventional studies.

Groups	Category	No. of papers	Percentage (%)
ITV Estimation method	*CV_1_*	51	94.4
	SD	4	7.4
	Range	1	1.9
	Min, Max	1	1.9
Data normalization	No normalization	49	90.7
	Log-transformation	3	5.6
	Cube-root transformation	1	1.9
	Min-max transformation	1	1.9
Minimum No. of trait samples	≤20	28	51.9
	[21, 50]	14	25.9
	[51, 100]	10	18.5
	>100	2	3.7

CV, SD, Range, Max, and Min represent the coefficient of variation, standard deviation, range, maximum, and minimum of sampled trait values, respectively. Detailed results of our literature survey about ITV estimation was given in **Table S1.**

Despite the widely appreciated merits of CV and the importance of accurate ITV estimation, it is less well-known that the most commonly used CV estimator (*CV*_1_= *σ_sample_*/*μ_sample_*) is biased (Sokal and Rohlf, 1995), which means that the real ITV of an entire population of a species cannot be accurately estimated from trait samples when the sample size is small (e.g., 10). Other CV estimators do exist (e.g., Bao, 2009) and may perform better than *CV_1_*, but no studies, to our knowledge, have compared the performance of these CV estimators using large empirical trait datasets, which may be considerably different from commonly used simulated data. For example, large trait datasets often contain few extreme values, and it is not clear if these values bias the ITV estimations and whether there are data normalization methods could reduce these biases.

In addition to choosing a suitable CV estimator and data normalization method, few studies quantify the minimum sample size needed for accurate estimation of ITV. As reviewed by Bastias et al. (2017), and here (**Table 1**), fewer than 50 individuals were sampled per species in more than 77.7% of reviewed studies. Although measuring traits on a very large number of individuals per species is not always feasible, such a small sample size may introduce large bias into the estimation of ITV and subsequent ecological inferences. For arbitrary trait distributions, the bias of *CV_1_* has an approximate and negative reciprocal relationship with sample size (Bao, 2009). This reveals the possibility that the true ITV in many contemporary studies is likely underestimated by *CV_1_*, and this underestimation may be more serious when the sample size is small (<10). Despite the potential importance of ITV in many ecological disciplines, no study has yet examined the effect of sample size. Moreover, because traits have various distributions and often take extreme values, whether there exist bounds for the minimum sample size of ITV estimation remains largely unknown.

Here, we attempt to address the above knowledge gaps and improve the estimation of ITV by answering the following three questions: (i) To what extent the current estimations of ITV are biased? (ii) Are there more accurate estimators and data transformations for estimating ITV? (iii) Are there prescriptive rules for identifying the minimum sample size needed for a given level of accuracy (here ±5%)? To answer these questions, we reviewed the previous literature and evaluated the performance of CV estimators using simulated and empirical trait datasets. Finally, we evaluated the accuracy of the best performing ITV estimator across various sample sizes.

## Materials and Methods

### Surveying Methods Commonly Used to Estimate ITV

To find the most commonly used methods for the quantification of ITV in recent literature, we searched the Web of Knowledge (http://thomsonreuters.com/web-of-knowledge/) in Sep. of 2019 for research articles containing the topic “intraspecific variation”, or “intraspecific variability”, or “individual variation” (including wildcard terms such as vari*, var*, and intra*) in the past 19 years, from 2000 to 2019. Studies that only used quantitative statistical tests such as ANOVA, Levene's test, and linear mixed-effects models to analyze or account for ITV were omitted, because comparing ITV among populations, species, studies, and regions requires knowledge about the absolute extent of ITV. We downloaded the full text of each remaining article and checked if and how ITV was calculated. Specifically, we summarized the absolute and relative number of studies that used each ITV estimation method, data normalization scheme, and the minimum sample sizes for each species in our literature survey (**Tables 1** and **S1**). These data revealed that a great majority (94%) of researchers used *CV_1_* (see below) as their estimator of ITV.

### Existing CV Estimators

There are four CV estimators (regression estimators of the CV such as described by Archana and Rao (2011) were ignored here). The first estimator, *CV_1_*, is

$$CV_{1}=\frac{\sigma_{sample}}{\mu_{sampe}},$$

where *σ_sample_* and *μ_sample_* are the standard deviation and mean of a given sample, respectively. This is the most commonly used estimator of CV despite its bias (Sokal and Rohlf, 1995). If the value of a trait follows the normal distribution, the bias of *CV_1_* is -*CV_1_*/(4*N*) (Sokal and Rohlf, 1995), where *N* is the sample size. Thus, the second estimator, *CV_2_*, is formulated by subtracting *CV_1_* with the bias term:

$$CV_{2}=CV_{1}+\frac{CV_{1}}{4N}.$$

Because *CV_2_* is derived under the assumption of a normal distribution, it may underperform when trait values are not normally distributed. Breunig (2001) proved mathematically that the bias of *CV_1_* also depends on the skewness and kurtosis of the distribution of trait values. Therefore, Breunig (2001) and Bao (2009) proposed two approximate estimators, *CV_3_* and *CV_4_* (also called Bao's CV estimator), respectively, which do not assume any specific trait distribution:

$$CV_{3}\approx CV_{1}\sqrt{1-\frac{CV_{1}}{N}(3CV_{1}-2\gamma_{1})},$$

$$CV_{4}\approx CV_{1}-\frac{CV_{1}^{3}}{N}+\frac{CV_{1}}{4N}+\frac{CV_{1}^{2}\gamma_{1}}{2N}+\frac{CV_{1}\gamma_{2}}{8N},$$

where *N* is the sample size, and γ_1_ and γ_2_ are the Pearson's measures of skewness and kurtosis of the trait sample distribution, respectively.

### New Composite CV Estimators

**Figure 1:**
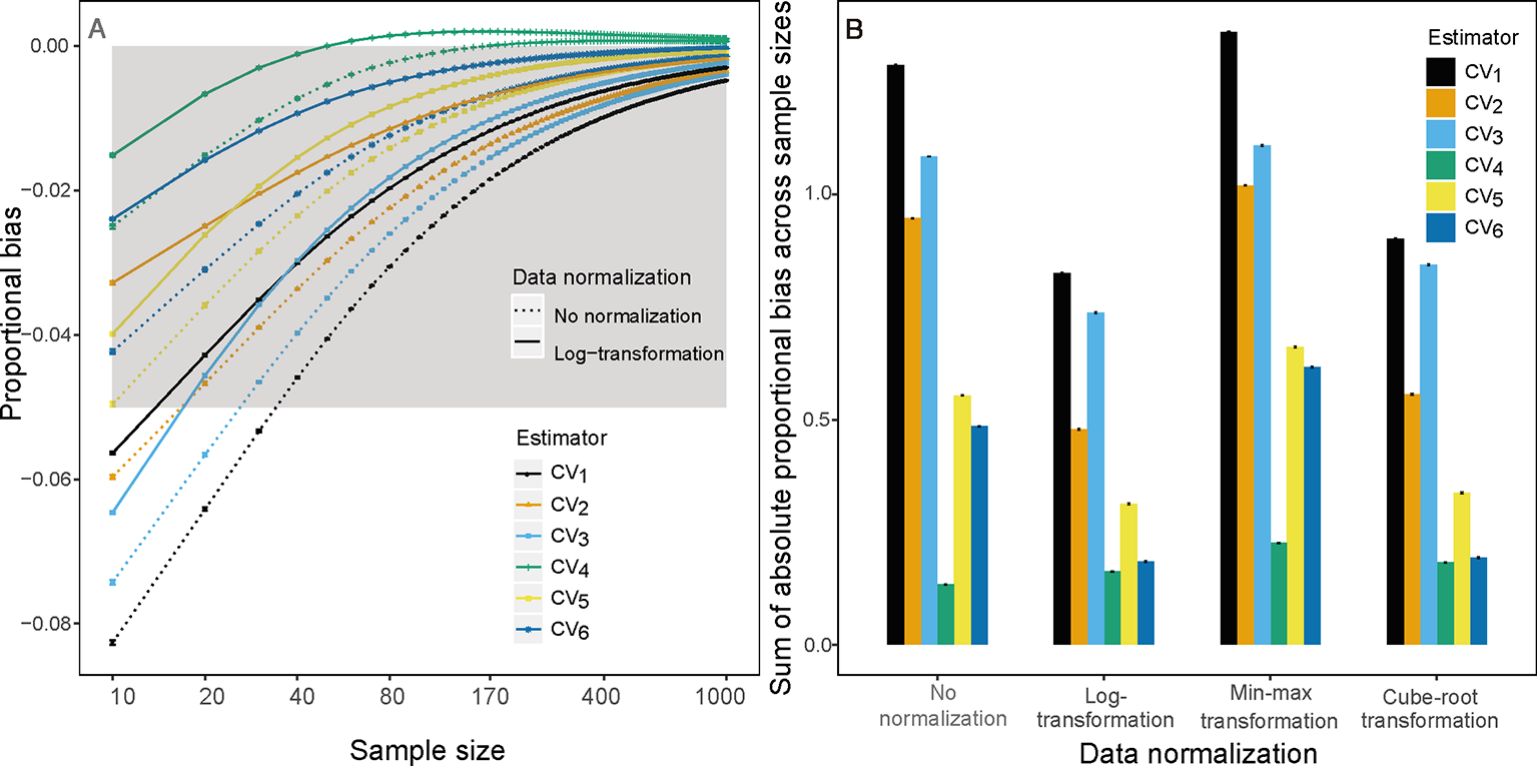
**(A)** Proportional bias on sample size *k*(*k*∈{10,20,30,…1,000}) based on raw (dotted line) and log-transform (solid line) simulated trait values and **(B)** sum of absolute proportional bias across all sample size for the *i* ^*th*^(*i*∈{1,2,3,4,5,6})CV estimator based on raw, log-transform, min-max transform and Cube-root transform simulated trait values. Gray area is the region in which the absolute mean proportion of bias is less than 0.05.

In our performance evaluation of CV estimators (below), we found that *CV_3_* and *CV_4_*—which are only approximate estimators of CV—often underestimate or overestimate the population CV in our simulation and empirical data (see **Figures 1A**, **S1A, S1E**, **S2I**, **S4A**, and **S4E**). Therefore, we propose four simple composite estimators, *CV*_5_, *CV_6_*, *CV*_7_, and *CV_8_*. *CV*_5_ and *CV*_7_ are defined as the arithmetic and geometric means of *CV_2_* and *CV_3_*, respectively, and *CV*_6_ and *CV*_8_ are defined as the arithmetic and geometric means of *CV_2_* and *CV_4_*, respectively.

$$\begin{array}{cc} CV_{5}=\frac{CV_{3}+CV_{4}}{2}, & CV_{6}=\frac{CV_{2}+CV_{4}}{2},\\ CV_{7}=\sqrt{CV_{3}CV_{4}}, & CV_{8}=\sqrt{CV_{2}CV_{4}}. \end{array}$$

We found *CV_7_* and *CV_8_* have almost identical performance compared to *CV_5_* and *CV_6_*, respectively, so we will only present the results of *CV_5_* and *CV_6_* in the remainder of the text. Note that *CV_3_* and *CV_4_* were derived without any assumption of trait value distribution, thereby their composite estimators, *CV_5_* and *CV_7_*, also make no assumptions of the true trait value distribution.

### Identifying the Minimum Sample Size

Accurate estimation of ITV not only depends on the choice CV estimator and data transformation, but also on sample size. Generally, the accuracy of any estimator increases with the sample size. Therefore, it is crucial to know how many samples are sufficient to reach a specific accuracy requirement in the estimation of ITV. Here, we defined the minimum sample size *k_i,min_* for estimator *i* as the minimum integer *k* that reaches ±5% accuracy of the ITV estimation. Because different traits of species may have different distributions, we calculated *k_i,min_* for each trait of each selected species separately.

### Performance Evaluation With Simulated Trait Data

To find the optimal CV estimator and data transformation, we evaluated the performance of the above estimators using simulated trait data. First, 200 trait pools were generated by simulation. Specifically, for each trait pool, 9,520 trait values were drawn from a gamma distribution with a shape parameter *β_1_* and a scale parameter *β_2_*, which were two independent random variables following a uniform distribution from 1 to 10 and from 5 to 30, respectively. The ranges of *β_1_* and *β_2_* were determined by fitting empirical trait value distributions using a gamma distribution for each trait of each species with abundance ≥100 in our trait plot (see details of the trait plot below). We chose the gamma distribution here because its domain, like empirical functional trait value, is always positive and its shape is flexible enough to model various distributions of traits. Additionally, empirical trait data often have a few (4.8% on average) extreme large values, which are defined here as trait values greater than three standard deviations from the mean. These extreme values may have large impacts on the accuracy of CV estimators. Therefore, we added 480 extreme large values to each simulated trait pool. The extent of the extreme value follows a truncated exponential distribution from mean+3*standard deviation of the trait pool to infinity and parameter *λ* equals 1. Note that we recognize that we cannot simulate all possible trait distributions observed in natural communities; here, we tried as best as possible to cover a large portion of the ranges of empirical trait distributions.

For each sample size *k*(*k*∈*N* and {*N*: 10, 15, 20, … 400}), *k* trait values were sampled from each simulated trait pool and *CV_i,k_* was calculated for each CV estimator *i*(*i*∈{1,2,3,4,5,6}). The mean CV, ${\bar{CV}}_{i,k}$, and its standard error were calculated from 9,999 replicates of sample size *k*. Then the bias of the *i* ^th^ estimator under sample size *k* was defined as $B(i,k)={\bar{CV}}_{i,k}-CV_{true}$, where *CV_true_* is the population CV that is known in our simulated data. Note that *CV_true_* is only required in our performance evaluation, and is not demanded in the estimation of ITV. The sign of *B*(*i*,*k*) indicates whether *CV_i,k_* under- or overestimates ITV. To facilitate estimator comparison, we calculated the proportion of bias (*PB*(*i*, *k*) = *B* (*i*, *k*)/*CV_true_*) for each sample size and estimator combination, and total absolute bias $\left(TPB(i)=\sum^{k}|B(i,k)|\right)$ for each estimator.

Our literature review further found that three types of data normalization methods are used in the estimation of ITV in conventional studies (**Table 1**). The first is min-max transformation defined as (*x*-*x_min_*)/*x_max_*, where *x* is the raw trait value and *x_min_* and *x_max_* are the minimum and maximum values of all sampled trait values, respectively. The second method is log-transformation with the natural logarithm base. For trait with values smaller than 1, 1 was added to all values of that trait before log-transformation. The last is cube-root transformation. To compare the effect of data normalizations on ITV estimation, *PB*(*i*,*k*) and *TPB*(*i*) were calculated for the raw trait data and three types of data normalizations for each CV estimator.

### Performance Evaluation With Empirical Trait Data

The performance of all the above estimators and data normalizations were also evaluated using three individual-based trait datasets, each representing a different geographic scale. For each dataset, we used the same sampling scheme as in the simulated data: 9,999 replicates were drawn with replacement for each sample size *k* from the observed trait values for each trait of each species, then *PB*(*i*, *k*) and *TPB*(*i*) were calculated for all *CV_i,k_*. Here, *CV_true_* was estimated by the value of *CV_1_* based on all individuals for each trait and each selected species.

The first dataset contained four traits: mean leaf area (MLA), specific leaf area (SLA), leaf dry mass content (LDMC) and individual height (Height). The dataset included 20,248 individuals (DBH≥1 cm or Height >1.3 m) belonging to 108 tree species in a 5ha subtropical forest plot, Tiantong (hereafter called Tiantong tree data), Zhejiang Province, China (Yan et al., 2018). This dataset represents ITV measurements at a local scale. Of the 108 species, we calculated ITV for seven species which had more than 400 individuals. We selected this abundance threshold because our performance criteria *PB*(*i*, *k*) and *TPB*(*i*, *k*), which depend on the *CV_true_*, can be estimated within ±0.55% accuracy by any of the above estimators when the sample size is larger than 400. The second trait dataset contained two traits (SLA and LDMC) from four tree species from 72 small (20 m × 20 m) plots in the whole Ningbo region (hereafter called Ningbo tree data), Zhejiang Province, China. It distributed along a 30 km gradient from seaside to inland—and is thus representative of regional scale data. The number of trait measurements for each trait in the Ningbo tree dataset was larger than 170. The third trait dataset contained five traits, head length (HL), interorbital distance (IOD), tympanum diameter (TYD), outer metacarpal tubercle width (OPTW), and tibia width (TW), of a mountain frog species (*Feirana quadrana*) from the mountain region covering Longmen-Qinling-Daba Mountains (hereafter called Mountain frog data), Central China. A total of 545 individuals were measured for each trait.

We implemented all of the above CV estimators in R functions, and made them available as an R package called “CV” (https://www.github.com/guochunshen/CV). All other performance tests and sample size analyses were performed in R environment (version 3.5.0, R Core Team, 2018).

## Results

**Figure 2:**
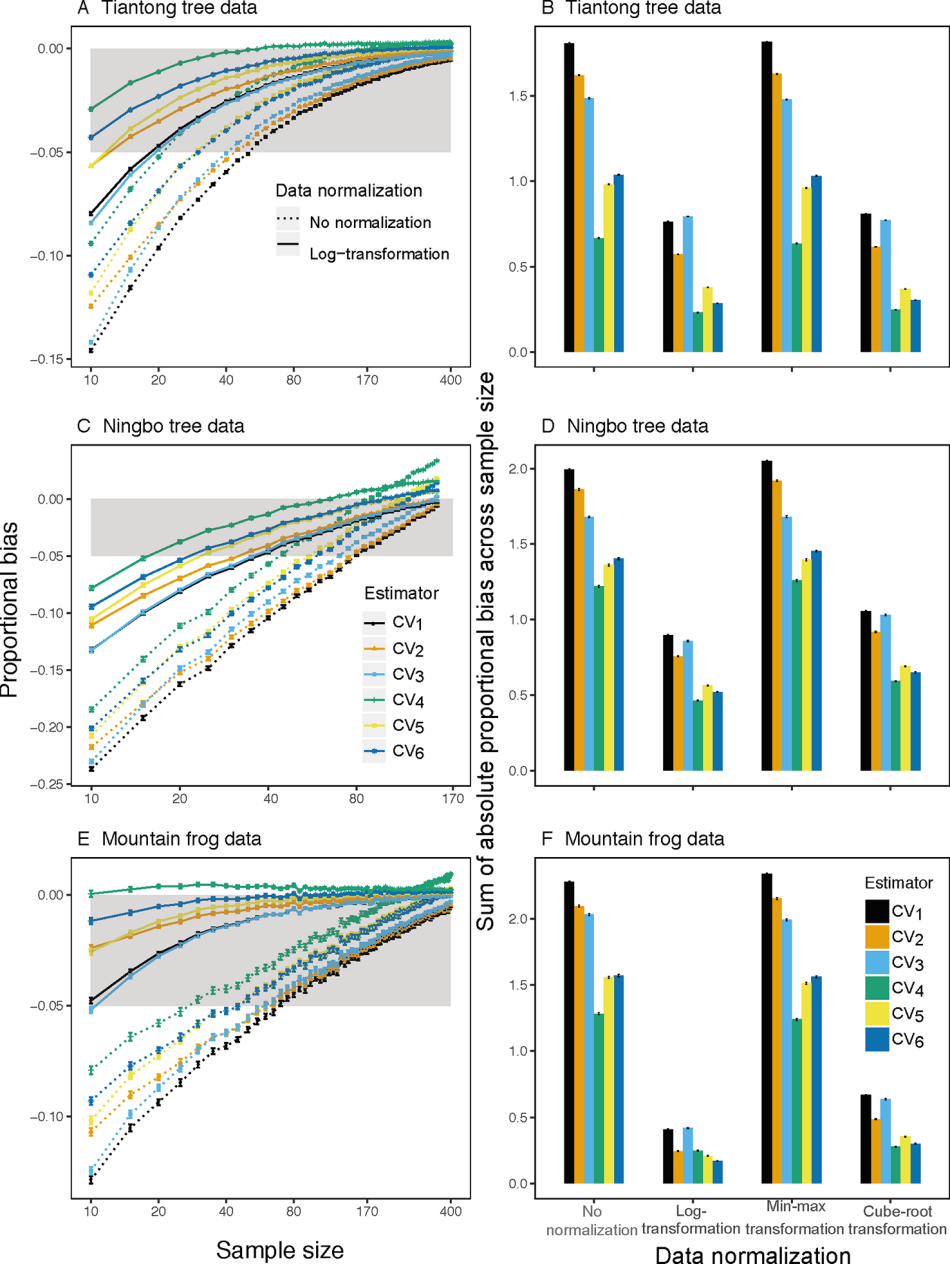
Mean proportion of bias (left panels) on sample size from 10 to 400 for all traits and species based on observed raw (dotted lines) and log-transform (solid lines) trait values and sum of absolute mean proportion of bias across all sample size (right panels) for the *i* ^*th*^(*i*∈{ 1,2,3,4,5,6, }) CV estimator based on observed raw, log-transform, min-max transform, and cube-root transform trait values in three individual-based trait datasets. Gray area is the region in which the absolute mean proportion of bias is less than 0.05.

Analyses of both simulated and empirical trait data showed that *CV_1_*, the most commonly used estimator of ITV, consistently underestimated ITV, particularly at small sample sizes (**Figures 1** and **2** and **S1–S4**). Furthermore, among all examined estimators, *CV_1_* had the largest proportional bias at each sample size (left panels in **Figures 1** and **2**) and total absolute bias across sample sizes (right panels in **Figures 1** and **2**). The averaged proportional bias of *CV_1_* increased with the extent of extreme values (**Figure S5**) and exceeded -23% of the true ITV at the sample size of 10 based on Ningbo tree data (black dotted lines in **Figure 2C**). For particular pairs of species and traits, the underestimations varied largely (**Figures S1–S4**)—reaching a maximum underestimation of ITV of 48.9% in LDMC of *Schima superba* in the Ningbo tree data (black dotted lines in **Figure S3K**).

**Figure 3:**
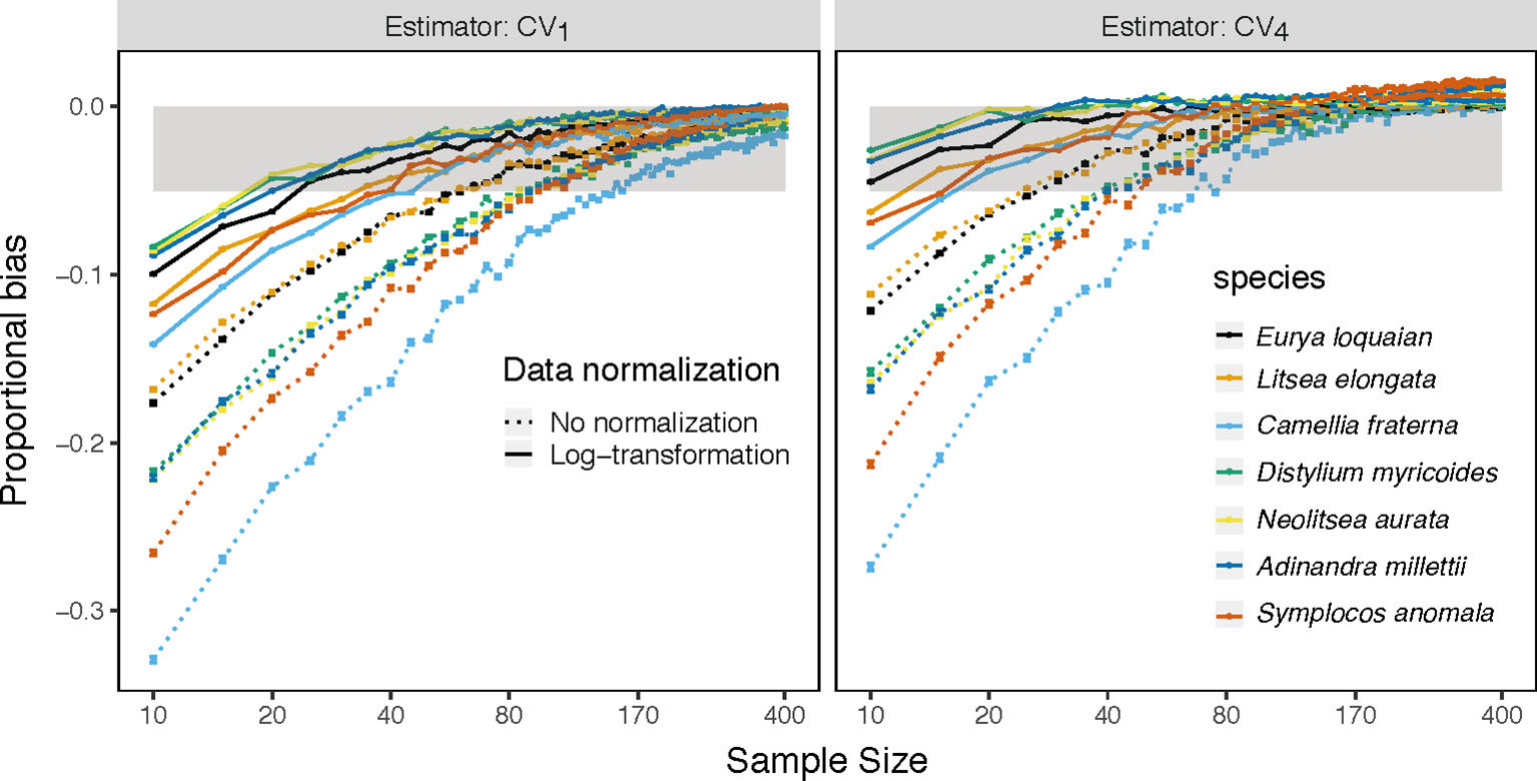
Mean proportion of bias of the *CV_1_* and the best estimator *CV_4_* on sample size *k*(*k*∈{10,15,20,…400}) for specific leaf area of each species (colored line) with abundance ≥ 400 based on the raw (dotted line) and log-transform (solid line) trait values in Tiantong tree data. Gray area is the region in which the absolute mean proportion of bias is less than 0.05.

This underestimation was substantially reduced by substituting *CV_1_* for other estimators and by log-transforming trait data. Among the examined estimators, *CV_4_* had the lowest bias using raw simulated and empirical trait datasets (bluish green dotted line and bar in **Figures 1** and **2**). The log-transformation further reduced the proportional bias of ITV for all estimators (solid lines in **Figures 1**–**3** and **S1–S4**), particularly at small sample sizes (<20), and especially for some species-trait pairs (e.g., SLA of *Camellia fraternal* in **Figure 3**). Pairwise Wilcoxon tests showed log-transformation significantly reduced mean proportional bias of *CV_4_* in Tiantong tree data (V = 506, P < 0.001), Ningbo tree data (V = 87, P < 0.005), and Mountain frog data (V = 529, P < 0.001). *CV_4_* was again found to be the most accurate estimator with log-transformed data, with one exception: *CV_6_* was the most accurate in the Mountain frog data. The cube-root transformation produced a similar, but relatively weaker reduction of ITV bias, and min-max normalization had no significant effects (right panels in **Figures 1** and **2**). Overall, *CV_4_* combined with log-transformed trait data was the most robust combination for accurate ITV estimation.

The *CV_1_* and *CV_4_* estimators allowed for the smallest minimum sample sizes to achieve a given level of accuracy (**Table 2** and **S2**). For the *CV_1_* estimator, the minimum sample size varied largely, ranged from 10 to 295, depending on the extent of the max extreme values for each trait (**Figure S6**). The *CV_4_* estimator could largely reduce the minimum sample sizes required for the same accuracy, but some species-trait pairs (e.g., *C. fraternal*-MLA) still required more than 100 samples. Finally, combined use of *CV_4_* with log-transformed trait data reduced the minimum sample size need to reach ±5% accuracy to just 20 samples for almost all examined traits and species.

**Table 2:** Minimum sample sizes of the most commonly used CV estimator *CV_1_* and our best-performed estimator *CV_4_* to reach ±5% accuracy for each trait (SLA, specific leaf area; MLA, mean leaf area; LDMC, leaf dry mass content, Height, individual height) of each species with abundance ≥400 based on the raw and log-transformed Tiantong tree data.

Species	Trait	Raw data		Log-transformed data	
		*ITV_1_*	*ITV_4_*	*ITV_1_*	*ITV_4_*
***Eurya loquaian***	SLA	65	*30*	25	*10*
	MLA	70	*30*	15	*10*
	LDMC	40	*20*	40	*20*
	Height	15	*10*	10	*10*
***Litsea elongata***	SLA	60	*25*	35	*15*
	MLA	20	*10*	10	*10*
	LDMC	35	*15*	40	*20*
	Height	25	*15*	10	*10*
***Camellia fraterna***	SLA	150	*70*	50	*20*
	MLA	295	*140*	25	*10*
	LDMC	35	*15*	40	*15*
	Height	15	*10*	10	*10*
***Distylium myricoides***	SLA	90	*40*	20	*10*
	MLA	45	*20*	10	*10*
	LDMC	40	*20*	45	*20*
	Height	10	*10*	10	*10*
***Neolitsea aurata***	SLA	85	*45*	20	*10*
	MLA	35	*15*	10	*10*
	LDMC	25	*10*	40	*15*
	Height	25	*15*	10	*10*
***Adinandra millettii***	SLA	95	*45*	25	*10*
	MLA	55	*25*	10	*10*
	LDMC	30	*15*	30	*10*
	Height	20	*10*	10	*10*
***Simplices anomala***	SLA	100	*50*	40	*20*
	MLA	25	*10*	15	*10*
	LDMC	20	*10*	45	*20*
	Height	55	*25*	10	*10*

## Discussion

By applying performance tests to both simulated and empirical data, we provided the first evidence that ITV quantified by *CV_1_* is often biased under sample sizes that are commonly applied (e.g., <50). When the sample size is around 10, the underestimation of ITV can exceed 48.9%. This pervasive underestimation of ITV by *CV_1_* has largely been ignored in previous studies and has therefore potentially misled subsequent ecological inferences when comparing ITVs among species. The bias of *CV_1_* was different among traits and species and these differences cannot be completely removed by using the same sample sizes, because the source of bias in *CV_1_* includes the skewness and kurtosis of the underlying trait distributions (Breunig, 2001), which generally differ among species and traits (**Figures S7–S9**). For example, using *CV_1_* and a sample size of 20, *Eurya loquaiana* appears to have significantly smaller ITV than *Symplocos setchuensis*; however, using the full empirical data yields exactly the opposite finding (**Figure S10A**). In this case, after log-transformation, both *CV_1_* and *CV_4_* correctly estimate the relatively magnitudes of ITV of the two species and *CV_4_* is very close to the true ITV (**Figure S10B**). Future studies of ITV based on CV should be aware of and address these biases. Below, we propose a few strategies to reduce bias based on our comparisons of estimators, data transformations, and samples sizes.

One easily adoptable solution to underestimation is to use a less biased and more robust CV estimator. Our results show that simply replacing the commonly used *CV_1_* for *CV_4_* substantially improved the accuracy of ITV estimation—sometimes by more than 58% (e.g., the average of height ITV of all species in Tiantong tree data). The higher accuracy of *CV_4_* results from its consideration the skewness and kurtosis of the trait distributions. However, *CV_4_* may overestimate ITV when the distribution of raw or log-transformed trait data is close to the normal distribution. *CV_4_* is an approximate form of CV, and therefore can only be truly unbiased when the underlying trait distribution is known exactly. Like *CV_1_*, *CV_2_* is derived under the assumption that trait values follow a normal distribution, which has no skewness and kurtosis. The contrasting behaviors of *CV_4_* and *CV_2_* allowed us to construct a simple composite estimator *CV_6_*, which was more accurate when the distribution of trait values is close to the normal distribution (**Figure S11**). In general, we suggest two alternative ITV estimators that apply in different contexts: *CV_4_* will be the least biased when trait data is non-normal—which we believe to be more common—otherwise, *CV_6_* is the best ITV estimator.

In additional to CV estimator selection, we recommend log-transforming raw trait data before using any ITV estimator. This normalization method can substantially improve the accuracy of ITV estimation because the raw trait data often have multiple extreme values that ITV estimators are very sensitive to. The log-transformation places less weight on these extreme large values, which results in a more robust estimation of ITV than the raw trait data. However, there were a few scenarios where log-transformation slightly increased the bias of ITV estimation (e.g., **Figures S1C** and **S3**) and additional caution is required when comparing ITVs among species based on log-transformed data. While log-transforming data can help reduce the skewness of data, it is more suited for comparison of ITV on the same log scale. If one feels more comfortable to quantify ITV at the scale of trait measurement or want to compare ITVs with conventional studies based on raw data, log-transformation, or any other data normalization, is not recommended. Instead, researchers should focus their efforts on increasing sample size (e.g., >140) to achieve comparable accuracy. In these cases, ITV can be variously biased, which makings comparisons of ITV challenging.

Next, although labor-intensive, increasing sample size will increase the accuracy of ITV estimation. If *CV_1_* is used, the minimum sample size needed to accurately estimate ITV is often larger than 50; although few conventional studies approach this number. Furthermore, minimum sample sizes required by *CV_1_* vary greatly among traits and species. These large differences likely reflect variation in traits’ distributions and extreme values, which is common in empirical data (Albert et al., 2010b). We showed that the minimum sample size of *CV_1_* depends on the extent of the extreme values, which suggests that the more extreme values present, the more samples may be required for the accurate estimation of ITV (**Figure S6**). The variation in the minimum sample size of *CV_1_* posits a unique practical challenge to determine the exact minimum sample size for a particular trait of a species. No ‘magic’ minimum sample size will work for all traits from different species. Fortunately, using *CV_4_* and log-transformation simultaneously can reduce sample sizes to about 20 while achieving ±5% accuracy in almost all examined combinations of species and traits. This minimum sample size can be more easily satisfied in the field of functional ecology.

Besides the above mentioned suggestions, there are other ways to improve the estimation of ITV. A smart sampling design other than the complete random sampling used in this study might be helpful. For example, the random sampling scheme may not be effective enough if a large amount of ITV is caused by heterogeneous abiotic environments and the individuals are not proportionally distributed among environmental types. In this situation, a sampling scheme that proportionally covers all types of environments may provide a more efficient representation of the true trait value distribution in the entire population and consequently may improve the accuracy and efficiency ITV estimation (e.g., Albert et al., 2010a; Baraloto et al., 2010 and van de Pol, 2012 but see Burton et al., 2017). Detailed exploration of these different sampling schemes is beyond the scope of this study but worth attention in future studies. Finally, there are other types of methods besides CV (e.g., mixed effect models) that could be used to quantify the relative extent of ITV. For example, if one's aim is to understand the consequences of ITV for community dynamics, directly modeling the trait’s distribution be more fruitful than simply expressing ITV with a single CV value. Detailed comparisons among these methods can be found in Mitchell and Bakker (2014).

In summary, we evaluated the bias in the estimation of ITV using the coefficient of variation with both simulated and empirical trait data. Our results clearly showed that the commonly used estimator *CV_1_* often underestimates ITV, and thus results of ITV studies based on *CV_1_* and small sample sizes should be interpreted with caution. The *CV_4_*, or Bao’s CV estimator, combined with log-transformed trait data can largely reduce this bias across many sample sizes, species, and traits. This combination can provide a more accurate tool for comparing trait variability within and among species and studies, facilitating a more robust inferences of the population dynamics (Abbott and Stachowicz, 2016), community assembly (Dibble and Rudolf, 2016; Mitchell et al., 2016) and ecosystem functioning (Barabás and D'Andrea, 2016), as well as facilitate an understanding of global climate change (Moran et al., 2016). More generally, the application of our results may also help reduce bias in any study that uses CV to estimate scientific phenomena, in other fields outside ecology.

## Data Availability Statement

The datasets generated for this study are available on request to the corresponding author.

## Author Contributions

GS designed this study. XW, EY, and JH collected the data. JY and JL analyzed all data with the assistance of GS. JY and YC created the figures. JY and GS wrote this manuscript, and all the co-authors provided some comments and suggestions for the final manuscript.

## Funding

This study was supported by the National Key Research and Development Program (2016YFC0503102 to GS) and the National Natural Science Foundation of China (31470487 and 31870404 to GS; 31670438 and 31270475 to EY; 31572290 and 31770568 to JH), as well as the ECNU Multifunctional Platform for Innovation (008).

## Conflict of Interest

The authors declare that the research was conducted in the absence of any commercial or financial relationships that could be construed as a potential conflict of interest.
