# Supplementary material for: Large Underestimation of Intraspecific Trait Variation and Its Improvements
Source: Front Plant Sci. 2020 Feb 13;11:53. doi: 10.3389/fpls.2020.00053 (PMC7031497; doi:10.3389/fpls.2020.00053)
Supplement: Supplementary file 1 [file DataSheet_1.pdf]

Supplementary Material

Large underestimation of intraspecific trait variation and its improvements

Jing Yang[1], Jiahui Lu[1], Yue Chen[1], Enrong Yan[1,2], Junhua Hu[3], Xihua Wang[1,2], Guochun Shen[1,2]*

[1]Tiantong National Station for Forest Ecosystem Research, School of Ecological and Environmental Sciences, East China Normal University, Shanghai, 200241 China

[2]Shanghai Institute of Pollution Control and Ecological Security, 1515 North Zhongshan Rd.(No.2), Shanghai, 200092, China

[3]Chengdu Institute of Biology, Chinese Academy of Sciences, Chengdu, 610041, China

*** Correspondence:** gcshen@des.ecnu.edu.cn.

1 Supplementary Figures and Tables

1.1 Supplementary Tables

Supplementary Table S1. Methods, their sample sizes and data normalization of studies that directly quantified intraspecific trait variation found in our literature survey. CV, SD, Range, Max and Min in the Method column mean coefficient of variation, standard deviation, range, maximum and minimum of sampled trait values, respectively. Number of samples in the third column was recoded as the number of individuals used in the estimation of ITV for each species.

Paper	Method	No. of samples	Data normalization
Malanson, DeRose, and Bekker, 2019	CV	88; 89	No
	CV	13	No
Bączek et al., 2019	CV	15	No
Crouzier et al., 2019	CV	30; 55	No
Martinez-Moral and Kannan, 2019	CV	515	Log-transformation
Astuti et al., 2019	CV	>50	No
Tanner and Bee, 2019	CV	44	No
Shen et.al., 2019	CV	>5	Min-max transformation
Zhang and Yu, 2018	CV	≥ 60	No
Yan et al., 2018	CV	> 25	No

Turin et al., 2018	CV	[31, 34]	No
Petruzzellis et al., 2017	CV	34	No
Helsen et al., 2017	CV	[15, 30]	No
Isaac et al., 2017	CV	4	No
Mitchell et al., 2017	CV	≥ 5	No
Vik et al., 2017	CV	8	No
Malyshev et al., 2016	CV	[3, 10]	No
Ainley et al., 2016	CV	10	No
Forey et al., 2016	SD	[3, 9]	No
Larrinaga et al., 2016	CV	15	Cube-root transformation
Kuppler et al., 2016	CV	97	No
Luo et al., 2016	SD, Range	Average‡ 87	No
Peck et al., 2015	Max, Min, the Wentworth Scale	≈ 15	No
Munilla and Guitián 2014	CV	54	No
Laforest-Lapointe 2014	CV	[214, 406]	No
Gagliardi et al., 2014	CV	[60, 500]	No
Cockrem et al., 2013	CV	[1, 48]	No
Azaza et al., 2013	CV	[20, 80]	No
Hajek et al., 2013	CV	[18, 20]	No
Villellas et al., 2013	CV	25	No
Wellstein et al., 2013	CV	30	No
Morozov et al., 2013	CV	[8, 64]	No
Fu et al., 2013	CV	[3, 10]	No
Curren et al., 2013	CV	[4, 16]	No
Møller et al., 2012	SD	[2, 349]	No
Zywiec et al., 2012	CV	[4, 15]	No
Garcia et al., 2011	CV	79	No
Bidau et al., 2011	CV	[5, 134]	No
Lazzari et al., 2010	CV	> 16	No
Marras et al., 2010	CV	≥ 16	No
Lobon-Cervia et al., 2010	CV	≥5	No
Byers et al., 2010	CV	[4, 24]	No
Albert et al., 2010	CV	≥ 63	No
Jung et al., 2010	CV	10	No
Cockrem et al., 2009	CV	[18, 54]	No
Violle et al., 2009	CV	100	No
Kumar et al., 2008	CV	27	No

Kleven et al., 2007	CV	[4, 10]	Log-transformation
Lu et al., 2007	CV	48	No
Laskemoen et al., 2007	CV	46	No
Blanck et al., 2006	CV	Average 44	Log-transformation
Lang et al., 2005	CV	21	No
Martins et al., 2005	CV	48	No
Kleunen et al., 2001	CV	40	No

†: [Minimum, Maximum] of sample sizes in a study; ‡: only an average sample size was given in the study.

Supplementary Table S2. Minimum sample sizes of the commonly used ITV estimator ITV_1 and our best estimator CV_4 for each trait (SLA: specific leaf area, LDMC: leaf dry mass content, HL: head length, IOD: interorbital distance, TYD: tympanum diameter, OPTW: outer metacarpal tubercle width, TW: tibia width) of four tree species and a frog species. Number of measurements for each trait were ≥ 150 for these four tree species in the raw and log-transform Ningbo tree data and ≥ 500 for the raw and log-transform Mountain frog data.

Type	Species	Trait	Raw data		Log-transform data	
			ITV_1	ITV_4	ITV_1	ITV_4
Tree	*Machilus thunbergii*	SLA	130	*80*	40	*15*
	Schima superba		30	*15*	15	*10*
	Symplocos sumuntia		80	*40*	20	*10*
	Neolitsea aurata		85	*55*	25	*15*
	Machilus thunbergii	LDMC	25	*10*	30	*15*
	Schima superba		130	*90*	105	*60*
	Symplocos sumuntia		25	*10*	20	*10*
	Neolitsea aurata		25	*15*	30	*15*
Frog	*Feirana quadrana*	HL	10	*10*	10	*10*
		IOD	20	*10*	15	*10*
		TYD	15	*10*	10	*10*
		OPTW	95	*50*	10	*10*
		TW	265	*155*	15	*10*

Supplementary Figures

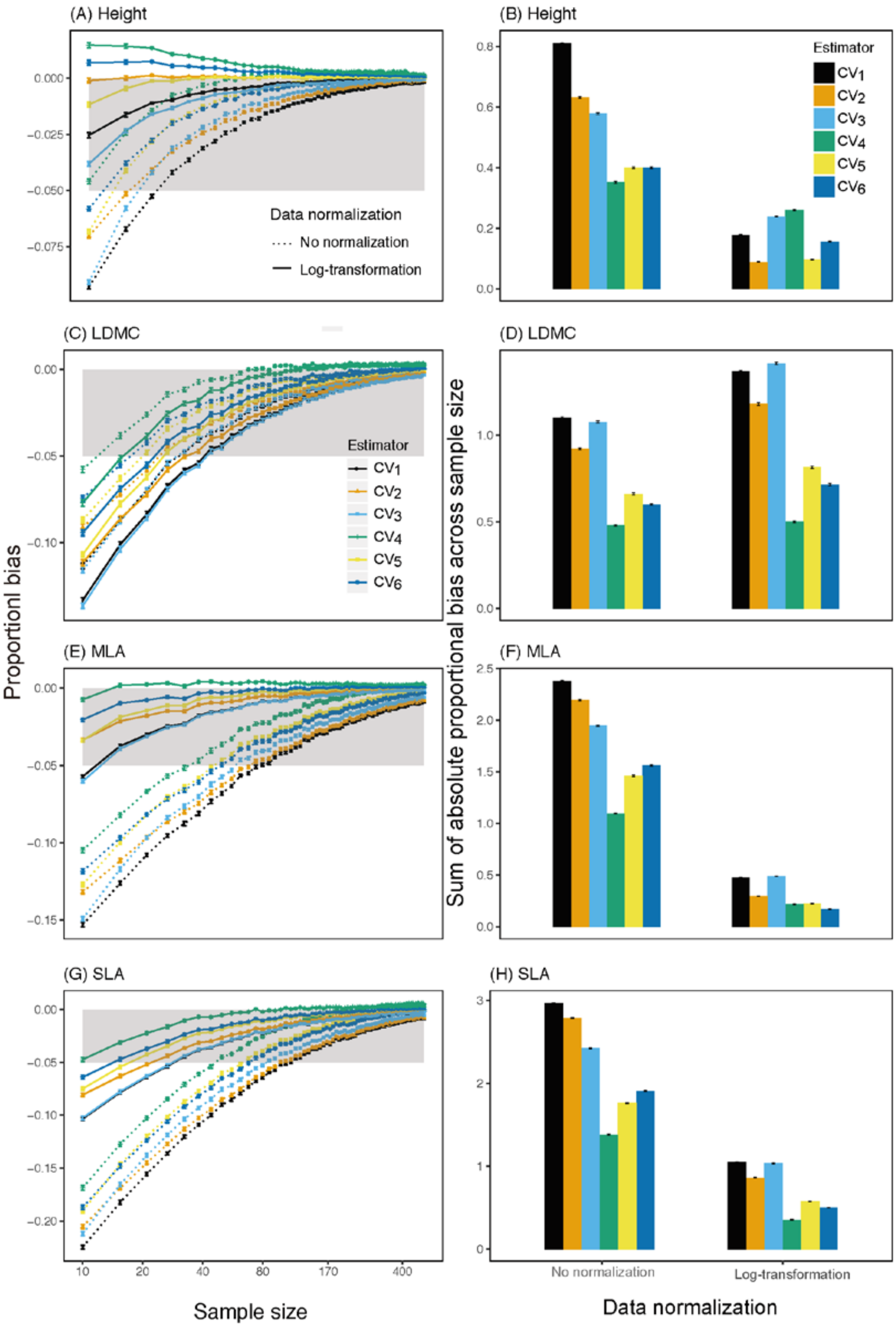

Supplementary Figure S1. Mean proportion of bias (left column) on sample size k ($k \in \{10, 15, 20, \dots 400\}$) for each trait of all species and sum of absolute mean proportion of bias (right column) across all sample size for the ith ($i \in \{1, 2, 3, 4, 5, 6\}$) ITV estimator based on observed trait values in the raw and log-transformed Tiantong tree data.

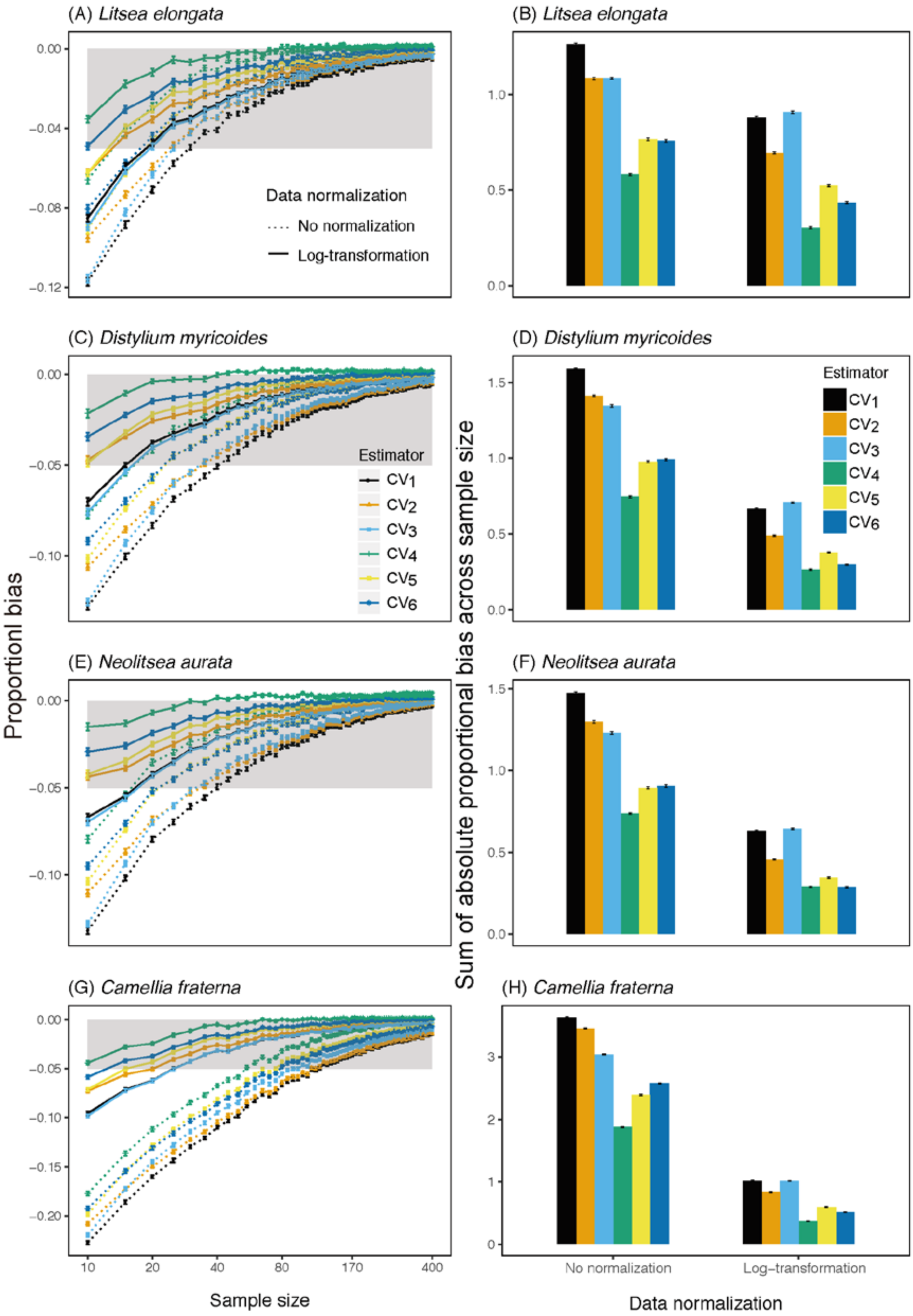

(A) Litsea elongata
(B) Litsea elongata
(C) Distylium myricoides
(D) Distylium myricoides
(E) Neolitsea aurata
(F) Neolitsea aurata
(G) Camellia fraterna
(H) Camellia fraterna
Data normalization
No normalization
Log-transformation
Estimator
CV1
CV2
CV3
CV4
CV5
CV6
Proportionl bias
Sum of absolute proportional bias across sample size
Sample size
Data normalization
No normalization
Log−transformation

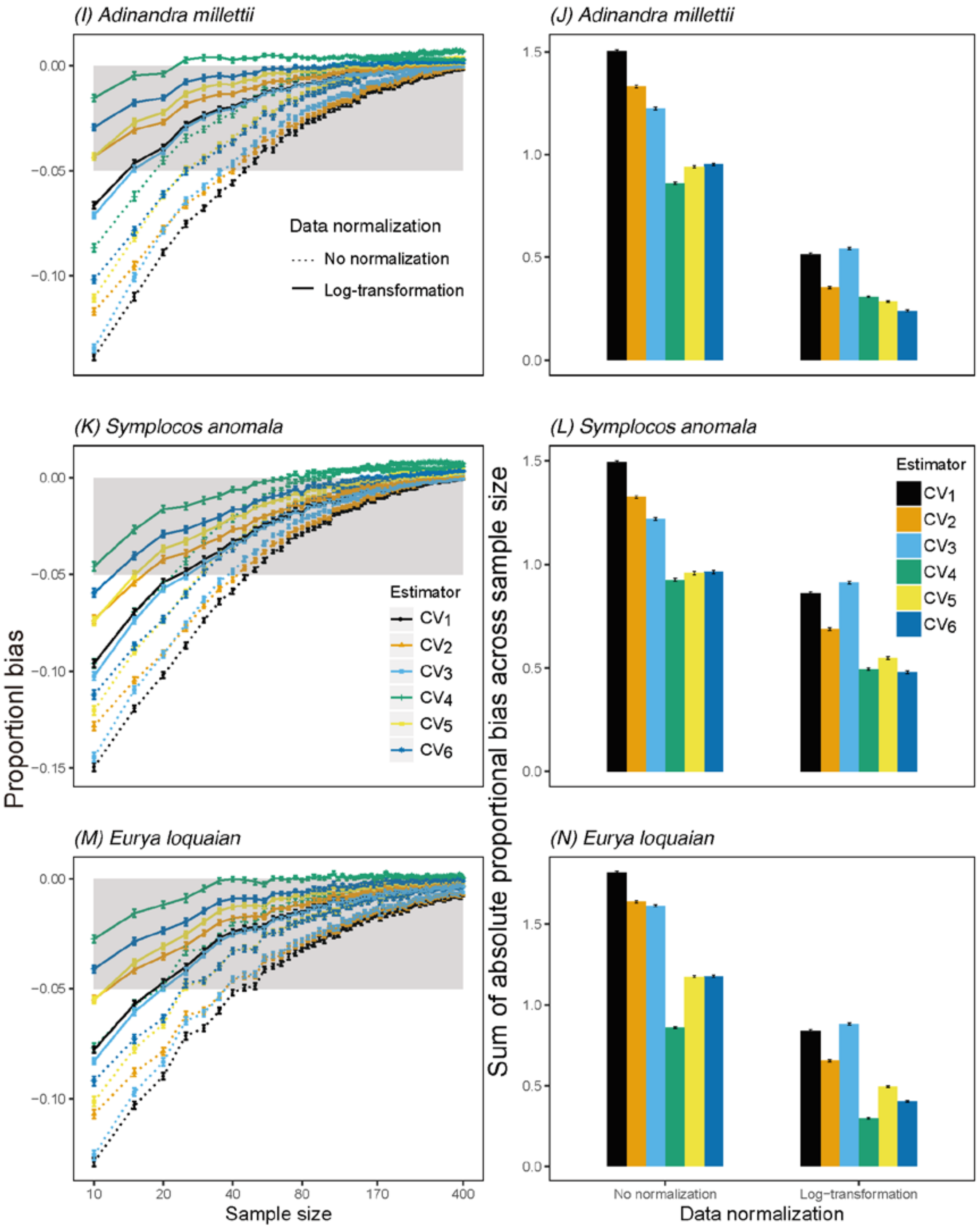

Supplementary Figure S2. Mean proportion of bias (left column) on sample size k ($k \in \{10, 15, 20, \ldots 400\}$) for each species of all traits and sum of absolute mean proportion of bias (right column) across all sample size for the ith ($i \in \{1, 2, 3, 4, 5, 6\}$) ITV estimator based on observed trait values in the raw and log-transformed Tiantong tree data.

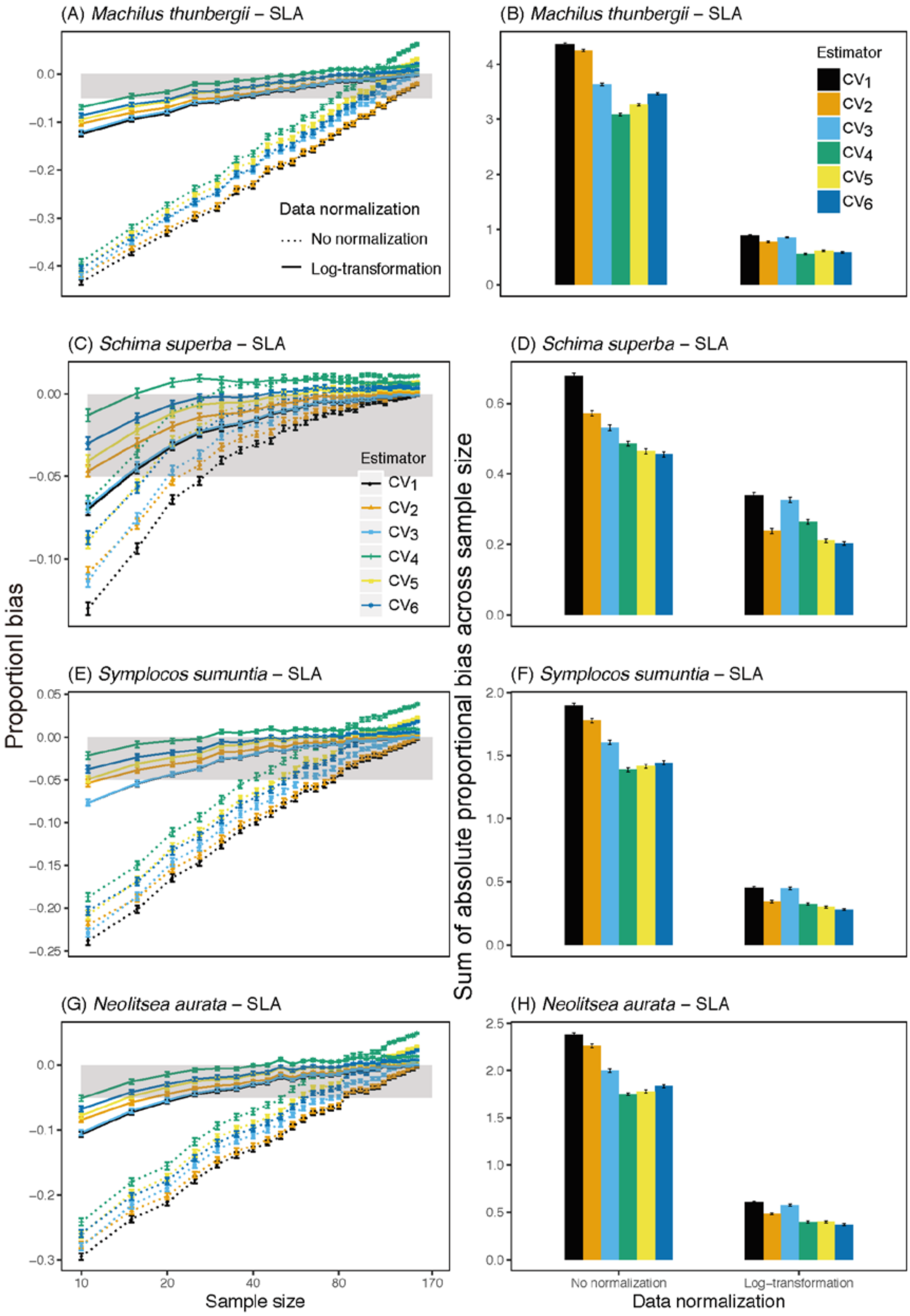

(A) Machilus thunbergii – SLA
(B) Machilus thunbergii – SLA
(C) Schima superba – SLA
(D) Schima superba – SLA
(E) Symplocos sumuntia – SLA
(F) Symplocos sumuntia – SLA
(G) Neolitsea aurata – SLA
(H) Neolitsea aurata – SLA
Data normalization
No normalization
Log-transformation
Estimator
CV1
CV2
CV3
CV4
CV5
CV6
Proportionl bias
Sum of absolute proportional bias across sample size
Sample size
Data normalization

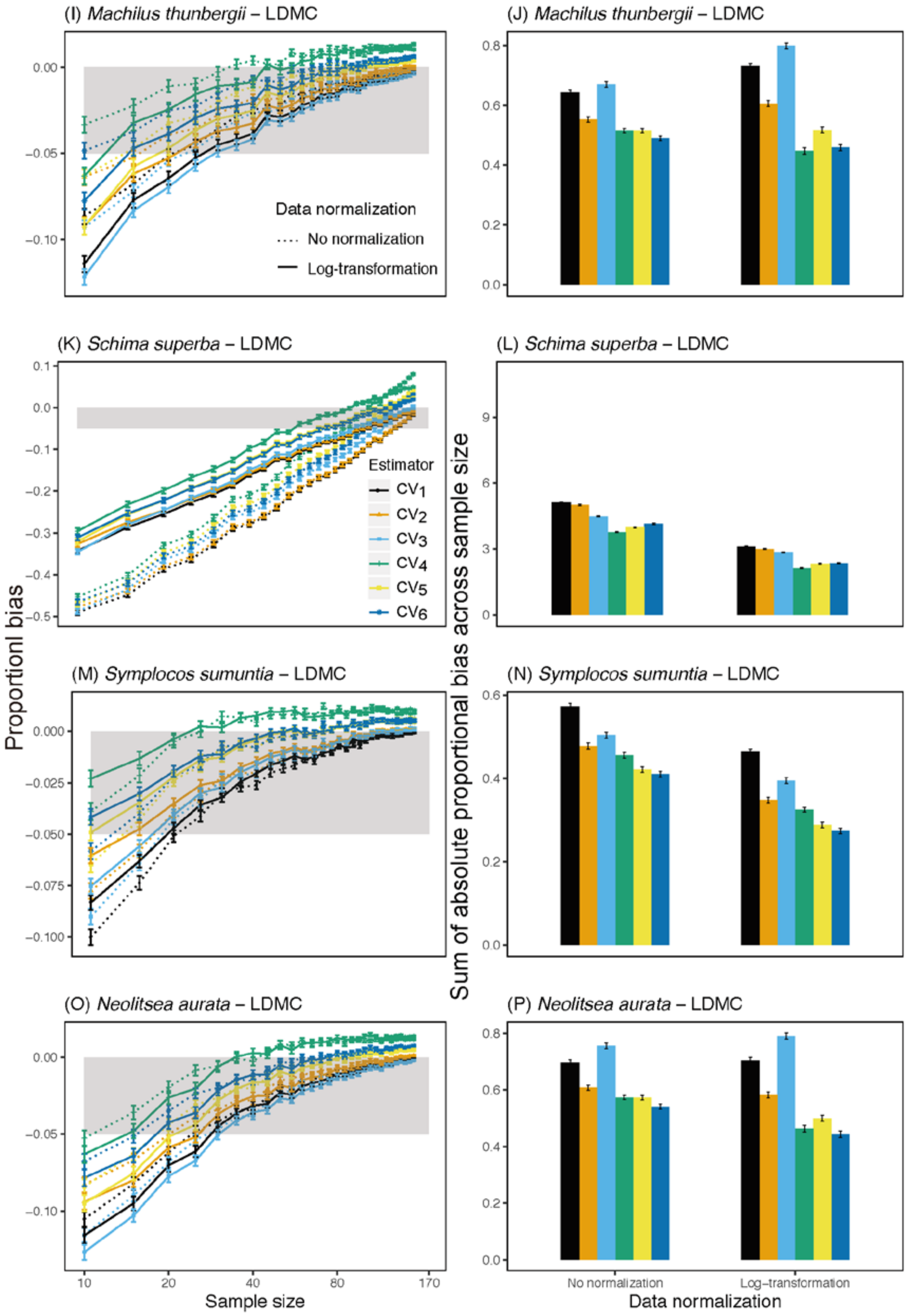

Supplementary Figure S3. Mean proportion of bias (left column) on sample size k ($k \in \{10, 15, 20, \dots 170\}$) and sum of absolute mean proportion of bias (right column) across all sample size for the ith ($i \in \{1, 2, 3, 4, 5, 6\}$) ITV estimator for each pair of species and trait in the raw and log-transformed Ningbo tree data.

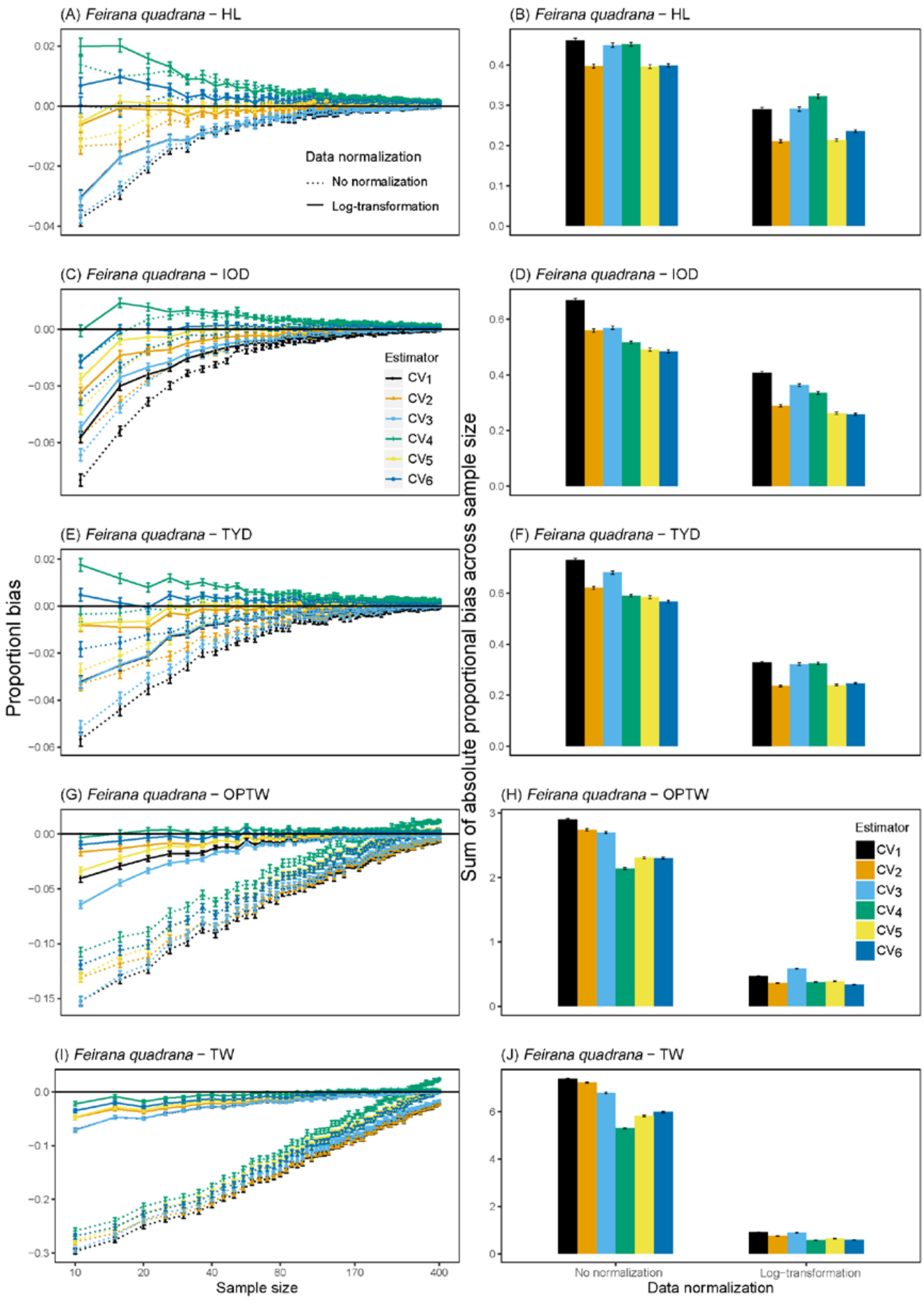

Supplementary Figure S4. Mean proportion of bias (left column) on sample size k ($k \in \{10, 15, 20, \ldots 400\}$) and sum of absolute mean proportion of bias (right column) across all sample size for the ith ($i \in \{1, 2, 3, 4, 5, 6\}$) ITV estimator for each pair of species and trait (HL: head length, IOD: interorbital distance, TYD: tympanum diameter, OPTW: outer metacarpal tubercle width, TW: tibia width) in the log-transformed Mountain frog data.

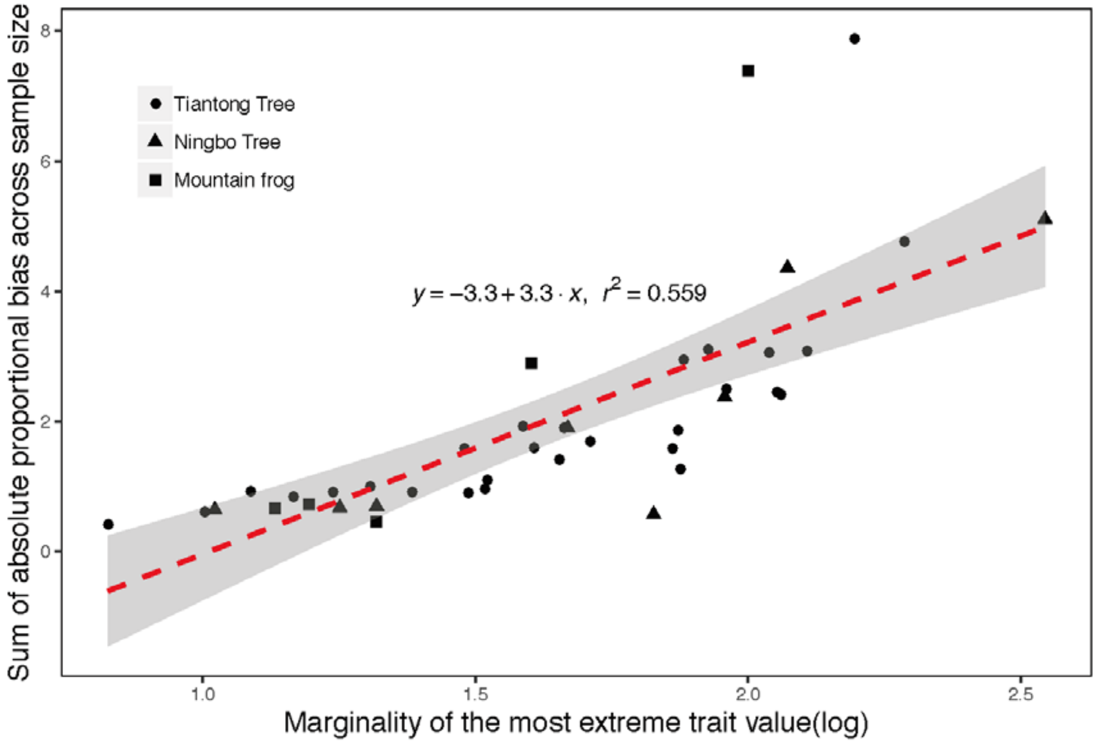

Supplementary Figure S5. Simple linear regression between the sum of absolute proportional bias across sample size and the marginality of the most extreme trait value (the most extreme trait value divided by the standard deviation and minus 1) of each trait of each species in all three real raw trait datasets. Red dotted line is the fitted regression line and grey area is the 95% confidence interval of the regression line.

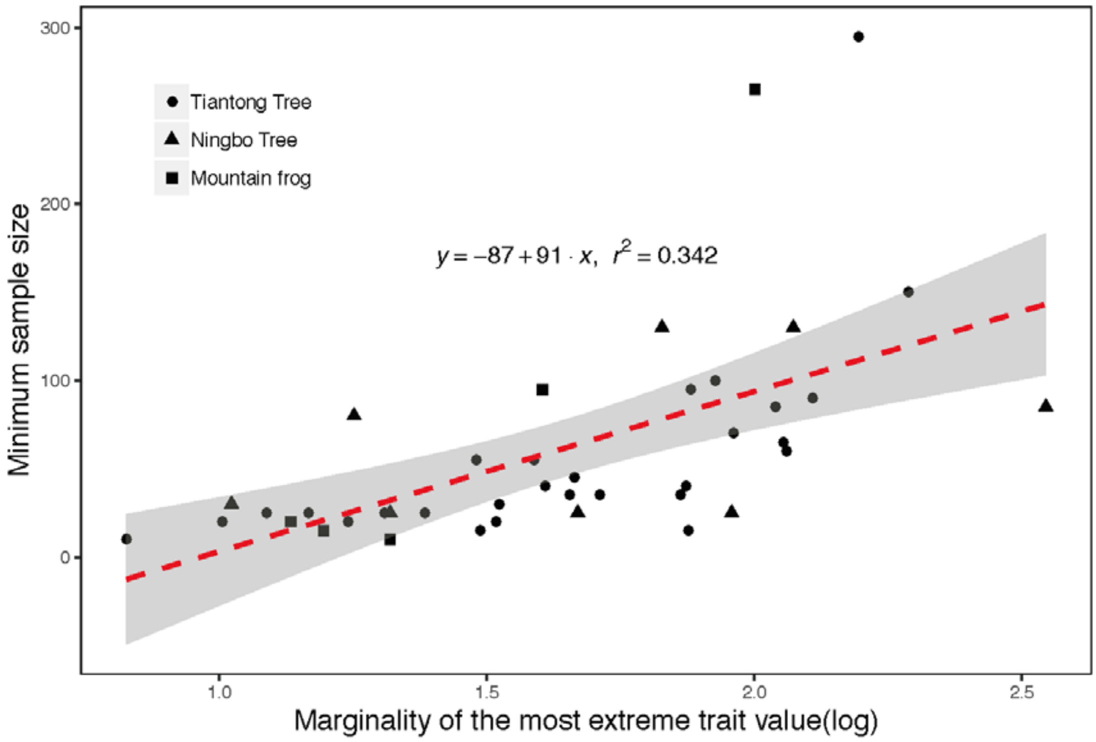

Supplementary Figure S6. Simple linear regression between the minimum sample size and the marginality of the most extreme trait value (the most extreme trait value divided by the mean trait value and minus 1) of each trait of each species in all three real trait datasets. Red dotted line is the fitted regression line and grey area is the 95% confidence interval of the regression line.

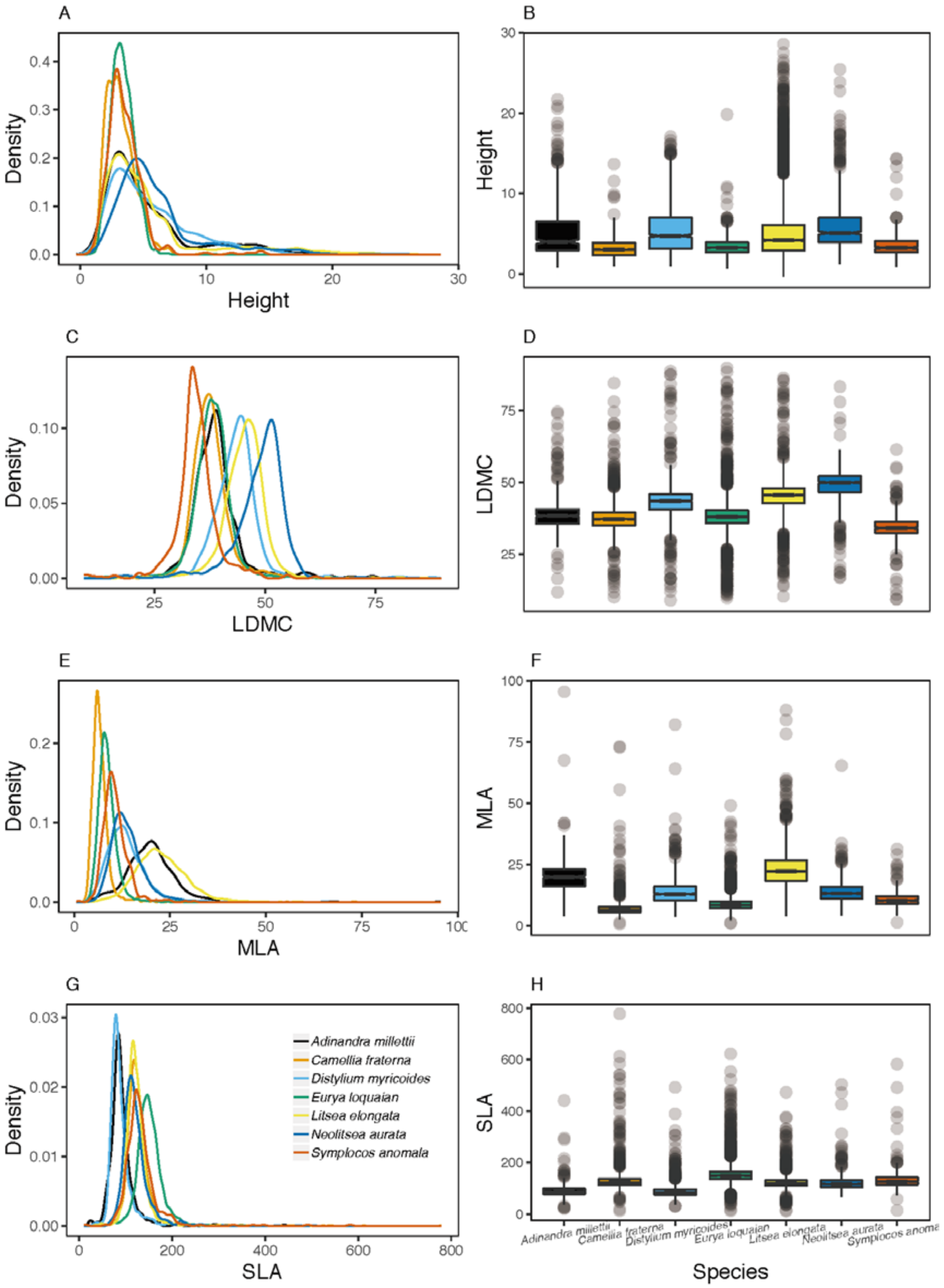

Supplementary Figure S7. Distributions (left column) and box plots (right column) of four trait values (mean leaf area (MLA), specific leaf area (SLA), leaf dry mass content (LDMC) and individual height (Height)) for species with abundance ≥ 400 in the Tiantong tree data.

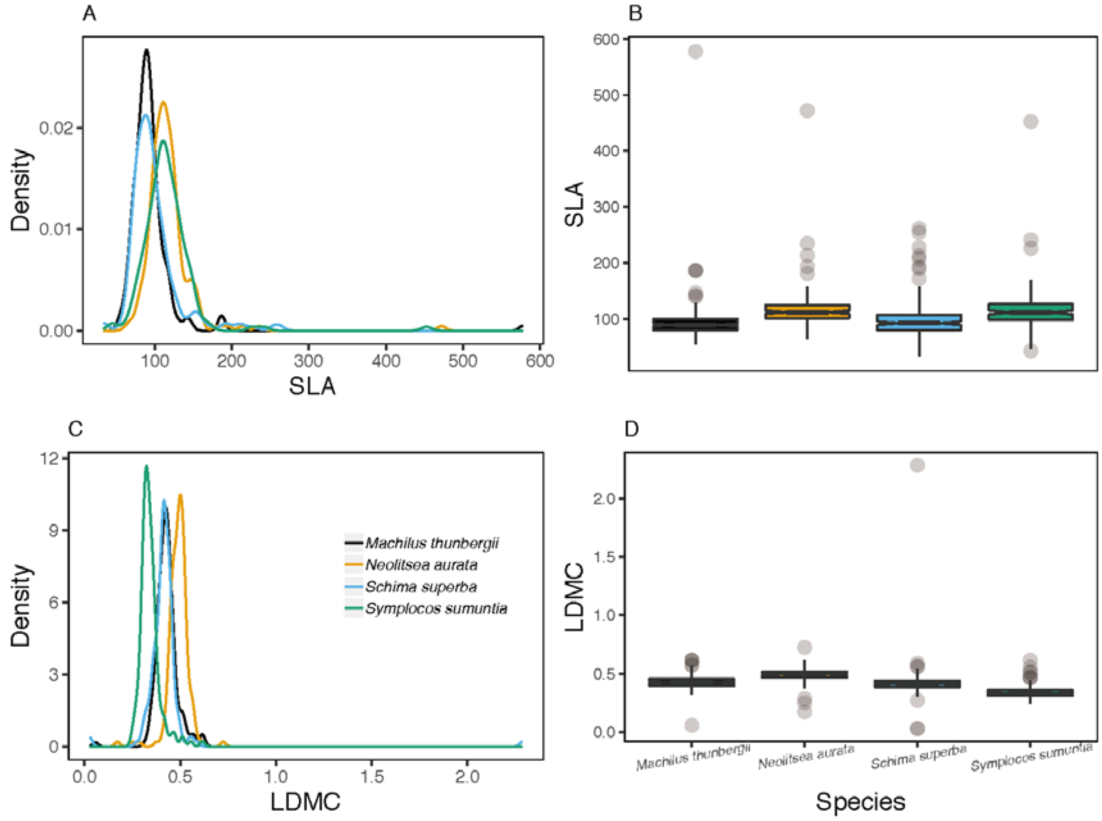

Supplementary Figure S8. Distributions (left column) and box plots (right column) of two trait values (specific leaf area (SLA) and leaf dry mass content (LDMC)) for species from the Ningbo tree data.

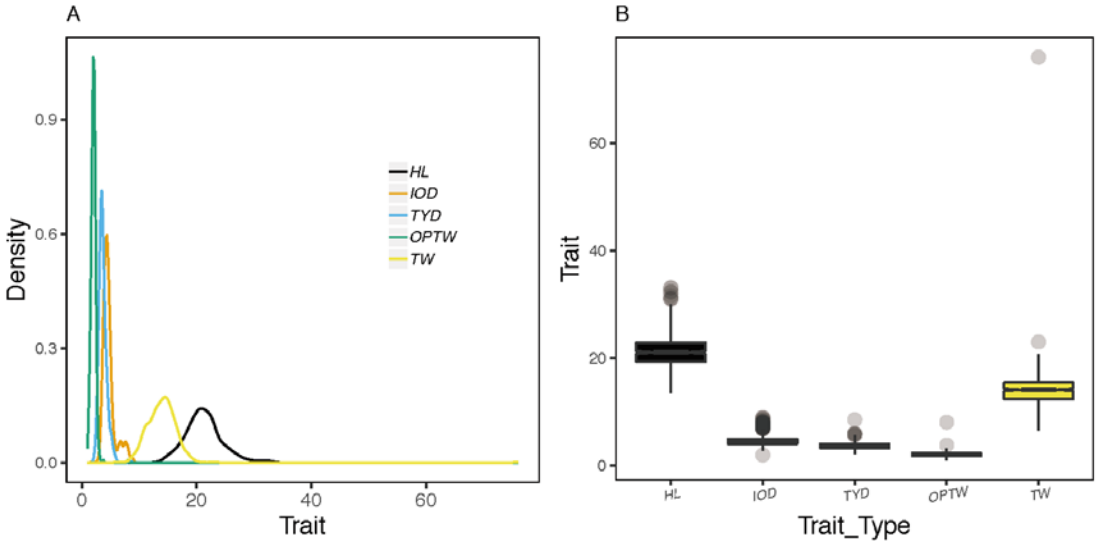

Supplementary Figure S9. (a) Distributions and (b) box plots of five trait values (head length (HL), interorbital distance (IOD), tympanum diameter (TYD), outer metacarpal tubercle width (OPTW) and tibia width (TW)) for a frog species from the Mountain frog dataset.

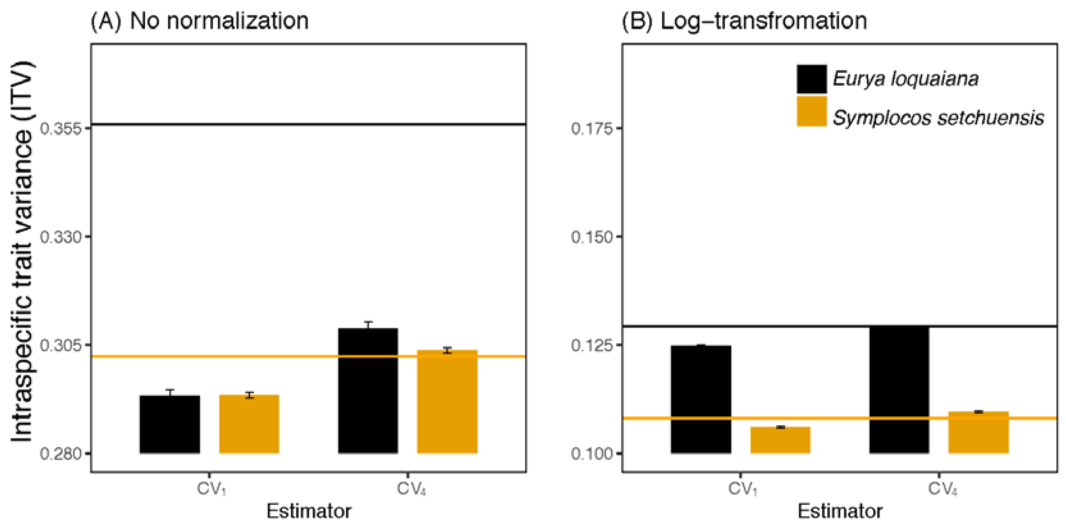

Supplementary Figure S10. A example of comparing intraspecific trait variations (ITVs) (±1 standard error bar) of mean leaf area between two species, *Eurya loquaiana* and *Symplocos setchuensis*, in the raw (A) and log-transform (B) Tiantong tree data. True ITVs (horizontal lines) are calculated by all recorded individuals of the two species, and is larger in *E. loquaiana* than in *S. setchuensis* Based on 20 randomly selected individuals, estimated ITVs by CV_4 can correctly reveal the order of tree ITVs between the two species, but CV_1 can't (left column). After log-transformation, both CV_1 and CV_4 both can be correctly reveal the order of ITVs between the two species (right column) and ITV estimated by CV_4 is quite close to the true ITVs.

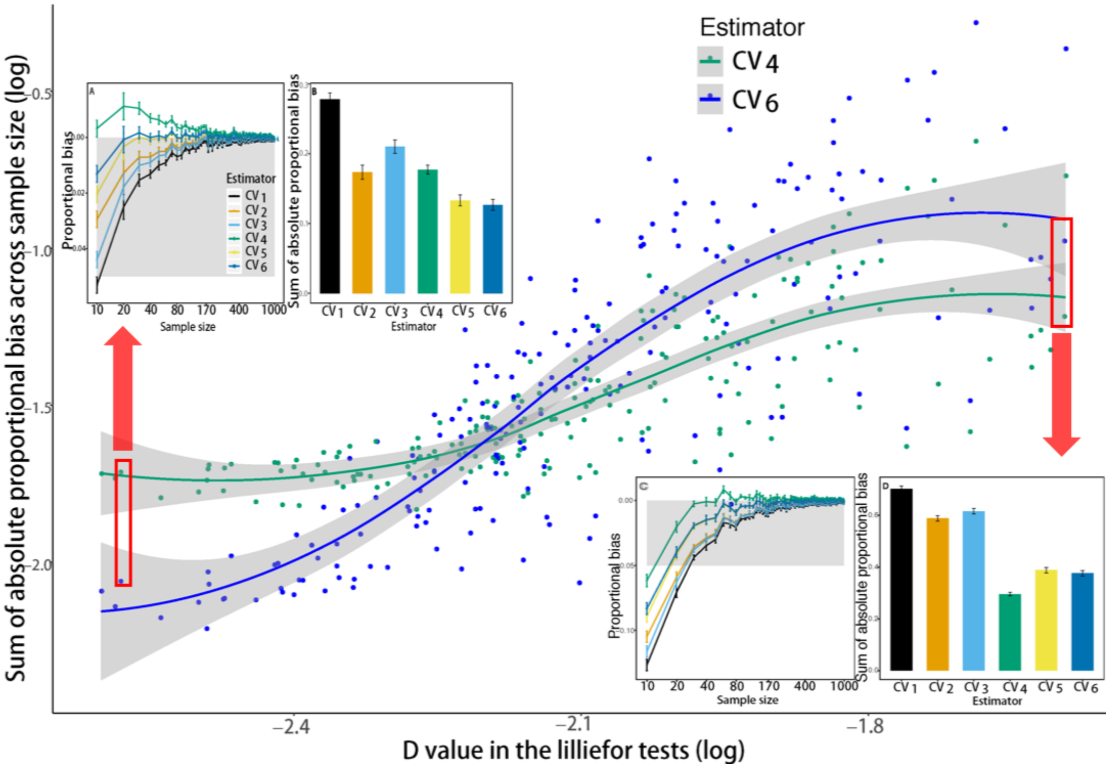

Supplementary Figure S11. Sum of absolute proportional bias across sample size for the ith ($i \in \{4, 6\}$) ITV estimator on D value in the Lilliefors Normality Test. of each simulated trait pool. The estimator CV_6 has the least total bias when the D value is smaller (A and B), but CV4 has the least total bias as D value in the Lilliefors Normality Test. is increasing (C and D).

References

Ainley, L.B., Verges, A. & Bishop, M.J. (2016) Congruence of intraspecific variability in leaf traits for two co-occurring estuarine angiosperms. *Oecologia,* **181,** 1041-1053.

Albert, C.H., Thuiller, W., Yoccoz, N.G., Soudant, A., Boucher, F., Saccone, P. & Lavorel, S. (2010) Intraspecific functional variability: extent, structure and sources of variation. *Journal of Ecology,* **98,** 604-613.

Astuti, G., Ciccarelli, D., Roma-Marzio, F., Trinco, A., and Peruzzi, L. (2019). Narrow endemic species Bellevalia webbiana shows significant intraspecific variation in tertiary CSR strategy. Plant Biosystems - An International Journal Dealing with all Aspects of Plant Biology 153 (1).

Azaza, M.S., Assad, A., Maghrbi, W. & El-Cafsi, M. (2013) The effects of rearing density on growth, size heterogeneity and inter-individual variation of feed intake in monosex male Nile tilapia Oreochromis niloticus L. *Animal,* **7,** 1865-1874.

Bączek, K., Pióro-Jabrucka, E., Kosakowska, O., and Węglarz, Z. (2019). Intraspecific variability of wild thyme (Thymus serpyllum L.) occurring in Poland. Journal of Applied Research on Medicinal and Aromatic Plants 12.

Bidau, C.J., Martí, D.A. & Baldo, D. (2011) Inter- and Intraspecific Geographic Variation of Body Size in South American Redbelly Toads of the Genus Melanophryniscus Gallardo, 1961 (Anura: Bufonidae). *Journal of Herpetology,* **45,** 66-74.

Blanck, A. & Lamouroux, N. (2006) Large-scale intraspecific variation in life-history traits of European freshwater fish. *Journal of Biogeography,* **34,** 862-875.

Byers, J.A. & Levi-Zada, A. (2010) Individual variation of (S)-4-methyl-3-heptanone in heads of braconid wasp, Leiophron uniformis, and Pogonomyrmex ants indicates costs of semiochemical production. *Chemoecology,* **21,** 35-44.

Cockrem, J.F. (2013) Individual variation in glucocorticoid stress responses in animals. *General & Comparative Endocrinology,* **181,** 45-58.

Cockrem, J.F., Barrett, D.P., Candy, E.J. & Potter, M.A. (2009) Corticosterone responses in birds: Individual variation and repeatability in Adelie penguins (Pygoscelisadeliae) and other species, and the use of power analysis to determine sample sizes. *General & Comparative Endocrinology,* **163,** 158-168.

Crouzier, M., Hug, F., Dorel, S., Deschamps, T., Tucker, K., and Lacourpaille, L. (2019). Do individual differences in the distribution of activation between synergist muscles reflect individual strategies? Experimental brain research 237 (3).

Curren, L.J., Weldele, M.L. & Holekamp, K.E. (2013) Ejaculate quality in spotted hyenas: intraspecific variation in relation to life-history traits. *Journal of Mammalogy,* **94,** 90-99.

Forey, E., Langlois, E., Lapa, G., Korboulewsky, N., Robson, T.M. & Aubert, M. (2016) Tree species richness induces strong intraspecific variability of beech (Fagus sylvatica) leaf traits and alleviates edaphic stress. *European Journal of Forest Research,* **135,** 707-717.

Fu, H., Yuan, G., Zhong, J., Cao, T., Ni, L. & Xie, P. (2013) Environmental and ontogenetic effects on intraspecific trait variation of a macrophyte species across five ecological scales. *PLoS One,* **8,** e62794.

Gagliardi, S., Martin, A.R., Filho, E.D.M.V., Rapidel, B. & Isaac, M.E. (2015) Intraspecific leaf economic trait variation partially explains coffee performance across agroforestry management regimes. *Agriculture Ecosystems & Environment,* **200,** 151-160.

Garcia, D., Ramos, A.J., Sanchis, V. & Marin, S. (2011) Intraspecific variability of growth and

patulin production of 79 Penicillium expansum isolates at two temperatures. *International Journal of Food Microbiology,* **151,** 195-200.
Hajek, P., Hertel, D. & Leuschner, C. (2013) Intraspecific variation in root and leaf traits and leaf-root trait linkages in eight aspen demes (Populus tremula and P. tremuloides). *Frontiers in Plant Science,* **4,** 415.
Helsen, K., Acharya, K.P., Brunet, J., Cousins, S.A.O., Decocq, G., Hermy, M., Kolb, A., Lemke, I.H., Lenoir, J., Plue, J., Verheyen, K., De Frenne, P. & Graae, B.J. (2017) Biotic and abiotic drivers of intraspecific trait variation within plant populations of three herbaceous plant species along a latitudinal gradient. *BMC Ecology,* **17,** 38.
Isaac, M.E., Martin, A.R., de Melo Virginio Filho, E., Rapidel, B., Roupsard, O. & Van den Meersche, K. (2017) Intraspecific Trait Variation and Coordination: Root and Leaf Economics Spectra in Coffee across Environmental Gradients. *Frontiers in Plant Science,* **8,** 1196.
Jung, V., Violle, C., Mondy, C., Hoffmann, L. & Muller, S. (2010) Intraspecific variability and trait-based community assembly. *Journal of Ecology,* **98,** 1134-1140.
Kleunen, M.V., Fischer, M. & Schmid, B. (2001) Effects of Intraspecific Competition on Size Variation and Reproductive Allocation in a Clonal Plant. *Oikos,* **94,** 515-524.
Kleven, O., Laskemoen, T., Fossoy, F., Robertson, R.J. & Lifjeld, J.T. (2008) Intraspecific variation in sperm length is negatively related to sperm competition in passerine birds. *Evolution,* **62,** 494-499.
Kumar, R.V., Tripathi, Y.K., Izhaki, I., Yadav, V.P. & Ahlawat, S.P. (2008) Intraspecific variation and interrelationships between morphology, nutritional content and enzymatic activity of Jatropha curcas L. *Current Science,* **95,** 239-243.
Kuppler, J., Hofers, M.K., Wiesmann, L. & Junker, R.R. (2016) Time-invariant differences between plant individuals in interactions with arthropods correlate with intraspecific variation in plant phenology, morphology and floral scent. *New Phytologist,* **210,** 1357-1368.
Laforest-Lapointe, I., Martínez-Vilalta, J. & Retana, J. (2014) Intraspecific variability in functional traits matters: case study of Scots pine. *Oecologia,* **175,** 1337-1348.
Lang, S.L.C., Iverson, S.J. & Bowen, W.D. (2005) Individual variation in milk composition over lactation in harbour seals (Phoca vitulina) and the potential consequences of intermittent attendance. *Canadian Journal of Zoology,* **83,** 1525-1531.
Larrinaga, A.R. & Guitián, P. (2016) Intraspecific variation in fruit size and shape in Corema album (Ericaceae) along a latitudinal gradient: from fruits to populations. *Biological Journal of the Linnean Society,* **118,** 940-950.
Laskemoen, T., Kleven, O., Fossøy, F. & Lifjeld, J.T. (2007) Intraspecific variation in sperm length in two passerine species, the Bluethroat Luscinia svecica and the Willow Warbler Phylloscopus trochilus. *Ornis Fennica,* **84,** 131-139.
Lazzari, V., Aguilar, J.P. & Michaux, J. (2010) Intraspecific variation and micro-macroevolution connection: illustration with the late Miocene genus Progonomys (Rodentia, Muridae). *Paleobiology,* **36,** 641-657.
Lobon-Cervia, J. (2010) Density dependence constrains mean growth rate while enhancing individual size variation in stream salmonids. *Oecologia,* **164,** 109-115.
Lu, Q., Zhong, W.-Q. & Wang, D.-H. (2007) Individual variation and repeatability of the aerobic performance in Brandt's voles (Lasiopodomys brandtii). *Journal of Thermal Biology,* **32,** 413-420.

Luo, Y.H., Liu, J., Tan, S.L., William, C.M., Wang, Y.H., Xu, K., Li, D.Z. & Gao, L.M. (2016) Trait-Based Community Assembly along an Elevational Gradient in Subalpine Forests: Quantifying the Roles of Environmental Factors in Inter- and Intraspecific Variability. *PLoS One,* **11,** e0155749.

Malyshev, A.V., Arfin Khan, M.A., Beierkuhnlein, C., Steinbauer, M.J., Henry, H.A., Jentsch, A., Dengler, J., Willner, E. & Kreyling, J. (2016) Plant responses to climatic extremes: within-species variation equals among-species variation. *Global Change Biology,* **22,** 449-464.

Marras, S., Claireaux, G., McKenzie, D.J. & Nelson, J.A. (2010) Individual variation and repeatability in aerobic and anaerobic swimming performance of European sea bass, Dicentrarchus labrax. *Journal Experimental Biology,* **213,** 26-32.

Martins, C.I.M., Schrama, J.W. & Verreth, J.A.J. (2005) The consistency of individual differences in growth, feed efficiency and feeding behaviour in African catfish Clarias gariepinus (Burchell 1822) housed individually. *Aquaculture Research,* **36,** 1509-1516.

Martinez-Moral, M.-P., and Kannan, K. (2019). How stable is oxidative stress level? An observational study of intra- and inter-individual variability in urinary oxidative stress biomarkers of DNA, proteins, and lipids in healthy individuals. Environment international 123. doi: 10.1016/j.envint.2018.12.009.

Mitchell, R.M., Wright, J.P. & Ames, G.M. (2017) Intraspecific variability improves environmental matching, but does not increase ecological breadth along a wet-to-dry ecotone. *Oikos,* **126**, 988-995

Møller, A.P. & Garamszegi, L.Z. (2012) Between individual variation in risk-taking behavior and its life history consequences. *Behavioral Ecology,* **23,** 843-853.

Morozov, A., Pasternak, A.F. & Arashkevich, E.G. (2013) Revisiting the role of individual variability in population persistence and stability. *PLoS One,* **8,** e70576.

Munilla, I. & Guitián, J. (2014) Long-term individual-level variation of reproductive features in Sorbus aucuparia, a fleshy-fruited tree. *Trees,* **28,** 1489-1496.

Peck, L.S., Thorne, M.A.S., Hoffman, J.I., Morley, S.A. & Clark, M.S. (2015) Variability among individuals is generated at the gene expression level. Ecology, 96(7), 2004-2014

Petruzzellis, F., Palandrani, C., Savi, T., Alberti, R., Nardini, A. & Bacaro, G. (2017) Sampling intraspecific variability in leaf functional traits: Practical suggestions to maximize collected information. *Ecology and Evolution,* **7,** 11236-11245.

Shen, G.C., Yan E.R., Bar-Massada, A., Zhang, J., Liu, H.M., Wang, X.H. and Xu, M.S. (2019) Species with moderate intraspecific trait variability are locally abundant within an environmentally heterogeneous subtropical forest. *Oecologia,* **190,** 629–637

Tanner, J. C., and Bee, M. A. (2019). Within-individual variation in sexual displays: signal or noise? Behavioral Ecology 30 (1).

Turin, R.A.F., Nali, R.C. & Prado, C.P.A. (2018) Intraspecific call variation in a Neotropical gladiator frog with a complex advertisement call. *Amphibia-Reptilia,* **39,** 31-39.

Vik, S.G., Dønnem, I. & Bøe, K.E. (2017) Individual variation in concentrate consumption rate of pregnant ewes. *Small Ruminant Research,* **151,** 22-25.

Villellas, J. & Garcia, M.B. (2013) The role of the tolerance-fecundity trade-off in maintaining intraspecific seed trait variation in a widespread dimorphic herb. *Plant Biology,* **15,** 899-909.

Violle, C., Castro, H., Richarte, J. & Navas, M.-L. (2009) Intraspecific seed trait variations and competition: passive or adaptive response? *Functional Ecology,* **23,** 612-620.

Wellstein, C., Chelli, S., Campetella, G., Bartha, S., Galiè, M., Spada, F. & Canullo, R. (2013) Intraspecific phenotypic variability of plant functional traits in contrasting mountain grasslands habitats. *Biodiversity and Conservation,* **22,** 2353-2374.

Yan, E.R., Zhou, L.L., Chen, H.Y.H., Wang, X.H. & Liu, X.Y. (2018) Linking intraspecific trait variability and spatial patterns of subtropical trees. *Oecologia,* **186,** 793-803.

Zhang, Z. & Yu, S. (2018) Potential tradeoffs between intraspecific and interspecific trait variations along an environmental gradient in a subtropical forest. *Journal of Forestry Research,* **3,** 1-10.

Żywiec, M., Delibes, M. & Fedriani, J.M. (2012) Microgeographical, inter-individual, and intra-individual variation in the flower characters of Iberian pear Pyrus bourgaeana (Rosaceae). *Oecologia,* **169,** 713-722.
