# Supplementary material for: Large Underestimation of Intraspecific Trait Variation and Its Improvements
Source: Front Plant Sci. 2020 Feb 13;11:53. doi: 10.3389/fpls.2020.00053 (PMC7031497; doi:10.3389/fpls.2020.00053)
Supplement: Supplementary file 2 [file DataSheet_2.pdf]

Large underestimation of ITV and its improvements

JingYang

2020/1/29

Load Packages

```
library(parallel)
library(ggplot2)
library(devtools)
library(easyGgplot2)
library(knitr)
library(scales)
library(nortest)
library(kableExtra)
```

Create core functions

CV

Calculate ITV of trait for a given sample size(N) by using four existing ITV estimators and our proposed two composite estimators **Note: We packed the first function including six estimators of ITV estimation into an R package called 'CV'**

```
CV <- function(N, trait) {
  if(N <= length(trait)){
    # sample N individuals from the traits values
    trait_sample <- sample(trait, N)
    # calcualte CV^2
    y_bar <- mean(trait_sample)
    s2_hat <- var(trait_sample)
    cv_2 <- s2_hat/y_bar^2
    cv_1 <- sqrt(cv_2)
    gamma_1 <- sum(((trait_sample - y_bar)/s2_hat^0.5)^3)/N
    gamma_2 <- sum(((trait_sample - y_bar)/s2_hat^0.5)^4)/N
    bias <- cv_2^(3/2)/N * (3 * cv_2^0.5 - 2 * gamma_1)
    bias2 <- cv_1^3/N - cv_1/4/N - cv_1^2 * gamma_1/2/N - cv_1 * gamma_2/8/N
    cv1 <- sd(trait_sample)/mean(trait_sample)
    cv2 <- (1 + 1/4/N) * cv1
    cv3 <- sqrt(cv_2 - bias)
    cv4 <- cv_1 - bias2
    cv5 <- mean(c(cv3, cv4))
    cv6 <- mean(c(cv2, cv4))
    return(c(cv1, cv2, cv3, cv4, cv5, cv6))
    }else{return(rep(NA,6))}
}
#Function--se
se=function(x){
  se=sd(x,na.rm=T)/sqrt(length(x[!is.na(x)]))
```

```
  return(se)
}
```

Simulated Trait CV

Calculate all cv bias of simulated trait values that were randomly drawn from a gamma distribution with parameters β1 and β2

```
Sim_CV <- function(N, trait_all) {
    # sample N individuals from the traits values
    # N_outliers <- round(rgamma(1,shape = shape_Noutliers, scale = scale_Noutliers)*15000)
    cv_true <- sd(trait_all)/mean(trait_all)
    #trait_sample <- rgamma(N, shape1, scale = shape2)
    trait_sample <- sample(trait_all, N)
    cv <- CV(N, trait_sample)
    cv_bias <- (cv - cv_true)/cv_true
    return(cv_bias)
}

# function - calculate mean cv_bias, cvbias_se, total_bias and totalbias_se of 200 simluted
 trait dataset
Sim_All_CVBias <- function(trait_all, Nsample, Single_Nrep, Cir_Nrep,Nshape){
    all_cv_bias <- matrix(NA, nrow = 6 * length(Nsample), ncol = Cir_Nrep)
    all_total_bias <- matrix(NA, nrow = 6, ncol = Cir_Nrep)
    for (c in 1:Cir_Nrep) {
        print(paste0("Cir", c))
        # Calculate cv bias using random simulated trait value
        cv <- list()
        for (i in 1:Nshape) {
            print(paste0("[", c, "-", i, "]", Sys.time()))
            all_cv <- unlist(mclapply(Nsample, function(x)
              apply(replicate(Nrep, Sim_CV(x, trait_all[i,])), 1, mean),
              mc.cores = 50))
            cv[[i]] <- data.frame(matrix(all_cv, nrow = 6))
            rownames(cv[[i]]) <- c("cv1", "cv2", "cv3", "cv4", "cv5", "cv6")
            colnames(cv[[i]]) <- Nsample
        }

        # Calculate mean cv bias of 200 random simulated results under each sample size
        cv_a <- c()
        cv_m <- c()
        for (n in 1:length(Nsample)) {
            for (k in 1:Nshape) {
                a <- cv[[k]][, n]
                cv_a <- c(cv_a, a)
            }
            cv_n <- matrix(cv_a, nrow = 6)
            cv_n <- apply(cv_n, 1, mean, na.rm=T)
            cv_m <- c(cv_m, cv_n)
        }

        all_cv_bias[, c] <- cv_m

        all_total_bias[, c] <- apply(abs(matrix(cv_m, nrow = 6, ncol = length(Nsample))), 1
, sum)
    }
```

```
    sim_cv_result <- data.frame(ITV = rep(c("ITV1", "ITV2", "ITV3", "ITV4", "ITV5", "ITV6")
, length(Nsample)),
                                sample_size = rep(Nsample, each = 6),
                                cv_bias = apply(all_cv_bias, 1, mean),
                                cvbias_se = apply(all_cv_bias, 1, se),
                                g = rep(1:6, length(Nsample)))

    sim_total_result <- data.frame(ITV = c("ITV1", "ITV2", "ITV3", "ITV4", "ITV5", "ITV6"),

                                   total_bias = apply(all_total_bias, 1, mean),
                                   cv_se = apply(all_total_bias, 1, se),
                                   g = 1:6)
    return(list(sim_cv_result,sim_total_result))
}
```

Real Trait CV

Calculate the average ITV and its bias by 9999 repetitions at different sample sizes for each species and each trait

```
Trait_CV <- function(data, species_name, trait_name, Nsample, Nrep, data_name) {
    cv_result <- data.frame()
    for (i in 1:length(species_name)) {
        spi <- data[data$sp == species_name[i], ]

        for (j in 1:length(trait_name)) {
            spi_traitj <- spi[, colnames(spi) == trait_name[j]]
            spi_traitj <- spi_traitj[!is.na(spi_traitj)]

            # Calculate the real ITV by using all trait data to in community
            cv_true <- sd(spi_traitj)/mean(spi_traitj)

            # Estimate ITV by using randomly selected sample trait data for different sampl
e size
            if (length(spi_traitj) >= max(Nsample)) {
                cv <- mclapply(Nsample, function(x) {
                  replicate(Nrep, CV(x, spi_traitj))
                }, mc.cores = 50)
            } else {
                # for the case that the maximal sample size is less than the maximal value
of Nsample
                Nsample <- seq(10, length(spi_traitj), 5)
                cv <- mclapply(Nsample, function(x) {
                  replicate(Nrep, CV(x, spi_traitj))
                }, mc.cores = 50)
            }

            mean <- list()
            cse <- list()
            for (k in 1:length(Nsample)) {
                mean[[k]] <- apply(cv[[k]], 1, mean)
                cse[[k]] <- apply(cv[[k]], 1, se)
            }

            spij_cv <- data.frame(species = species_name[i],
                                  trait = trait_name[j],
                                  sample_size = rep(Nsample, each = 6),
```

```
                                  g = rep(1:6, length(Nsample)),
                                  cv = unlist(mean), cv_true = cv_true,
                                  cv_bias = (unlist(mean) - cv_true)/cv_true,
                                  cv_se = unlist(cse),
                                  cvbias_se = unlist(cse)/cv_true,
                                  data_name = data_name)
            cv_result <- rbind(cv_result, spij_cv)
        }
    }
    return(cv_result)
}
```

All/Species/Trait CVBias

```
# Function: summary results of all species and all traits
All_CVBias <- function(result,Nsample,name){

  cv_bias <- matrix(NA, nrow = 6*length(Nsample), ncol = length(result))
  total_bias <- matrix(NA, nrow = 6, ncol = length(result))
  # rownames(cv_bias) <- paste0(rep(1:6, length(Nsample)),"_",rep(Nsample,each=6))
  # rownames(total_bias) <- 1:6

  for(i in 1:length(result)){
    resulti <- result[[i]]
    bias <- tapply(resulti$cv_bias,list(resulti$g,resulti$sample_size),mean,na.rm=T)
    cv_bias[,i] <- as.numeric(bias)
    total_bias[,i] <- apply(abs(bias),1,sum)
  }

all_cv_bias <- data.frame(g=rep(1:6,length(Nsample)),
                          sample_size=rep(Nsample,each=6),
                          cv_bias=apply(cv_bias,1,mean),
                          cvbias_se=apply(cv_bias,1,se),
                          data_name=name)

all_total_bias <- data.frame(g=1:6,
                            total_bias=apply(total_bias,1,mean),
                            cv_se=apply(total_bias,1,se),
                            data_name=name)

return(list(all_cv_bias=all_cv_bias,all_total_bias=all_total_bias))
}

### summary results of each species for all traits
Species_CVBias <- function(result,Nsample,species,name){

  sp_cv_bias <- matrix(NA, nrow = 6*length(Nsample)*length(species), ncol = length(result))
  sp_total_bias <- matrix(NA, nrow = 6*length(species), ncol = length(result))
  # rownames(sp_cv_bias) <- paste0(rep(tiantong_species_name,each=6*length(tiantong_Nsample
)),"_",
  #                              rep(1:6,length(tiantong_species_name)*length(tiantong_Nsam
ple)),"_",
  #                              rep(rep(tiantong_Nsample,each=6),length(tiantong_species_n
ame)))
  # rownames(sp_total_bias) <- paste0(tiantong_species_name[n],1:6)
```

```
  for(i in 1:length(result)){
    resulti <- result[[i]]
    sp_bias <- tapply(resulti$cv_bias,list(resulti$g,resulti$sample_size,resulti$species),m
ean,na.rm=T)
    sp_cv_bias[,i] <- as.numeric(sp_bias)
    for (n in 1:length(species)){
      sp_nrow <- ((n-1)*6+1):(n*6)
      sp_total_bias[sp_nrow,i] <- apply(abs(sp_bias[,,n]),1,sum)
    }
  }

  sp_cv_bias <- data.frame(species=rep(species,each=6*length(Nsample)),
                           g=rep(1:6,length(species)*length(Nsample)),
                           sample_size=rep(rep(Nsample,each=6),length(species)),
                           cv_bias=apply(sp_cv_bias,1,mean),
                           cvbias_se=apply(sp_cv_bias,1,se),
                           data_name=name)

   sp_total_bias <- data.frame(species=rep(species,each=6),
                               g=rep(1:6,length(species)),
                               total_bias=apply(sp_total_bias,1,mean),
                               cv_se=apply(sp_total_bias,1,se),
                               data_name=name)

return(list(sp_cv_bias=sp_cv_bias,sp_total_bias=sp_total_bias))
}

### summary results each trait of all species
Trait_CVBias <- function(result,Nsample,trait,name){

  trait_cv_bias <- matrix(NA, nrow = 6*length(Nsample)*length(trait), ncol = length(result)
)
  trait_total_bias <- matrix(NA, nrow = 6*length(trait), ncol = length(result))
  # rownames(trait_cv_bias) <- paste0(rep(tiantong_trait_name,each=6*length(tiantong_Nsampl
e)),"_",
  #                              rep(1:6,length(tiantong_trait_name)*length(tiantong_Nsampl
e)),"_",
  #                              rep(rep(tiantong_Nsample,each=6),length(tiantong_trait_nam
e)))
  # rownames(trait_total_bias) <- paste0(tiantong_trait_name[n],1:6)

  for(i in 1:length(result)){
    resulti <- result[[i]]
    resulti$trait <- factor(resulti$trait, levels = trait)
    trait_bias <- tapply(resulti$cv_bias,list(resulti$g,resulti$sample_size,resulti$trait),
mean,na.rm=T)
    trait_cv_bias[,i] <- as.numeric(trait_bias)
    for (n in 1:length(trait)){
      trait_nrow <- ((n-1)*6+1):(n*6)
      trait_total_bias[trait_nrow,i] <- apply(abs(trait_bias[,,n]),1,sum)
    }
  }

  trait_cv_bias <- data.frame(trait=rep(trait,each=6*length(Nsample)),
                              g=rep(1:6,length(trait)*length(Nsample)),
                              sample_size=rep(rep(Nsample,each=6),length(trait)),
                              cv_bias=apply(trait_cv_bias,1,mean),
                              cvbias_se=apply(trait_cv_bias,1,se),
```

```
                              data_name=name)

   trait_total_bias <- data.frame(trait=rep(trait,each=6),
                                  g=rep(1:6,length(trait)),
                                  total_bias=apply(trait_total_bias,1,mean),
                                  cv_se=apply(trait_total_bias,1,se),
                                  data_name=name)

return(list(trait_cv_bias=trait_cv_bias,trait_total_bias=trait_total_bias))
}


### total cv bias for each species and each trait
Spt_CVBias <- function(result,Nsample,species,trait,name){

  spt_cv_bias <- matrix(NA, nrow = 6*length(Nsample)*length(trait)*length(species), ncol =
length(result))
  spt_total_bias <- matrix(NA, nrow = 6*length(trait)*length(species), ncol = length(result
))

  for(i in 1:length(result)){
    resulti <- result[[i]]
    spt_total_bias[,i] <- as.numeric(tapply(abs(resulti$cv_bias),
                                            list(resulti$g,resulti$trait,resulti$species),s
um,na.rm=T))
  }

  spt_total_bias <- data.frame(species=rep(species,each=6*length(trait)),
                               trait=rep(rep(trait,each=6),length(species)),
                               g=rep(1:6,length(trait)*length(species)),
                               total_bias=apply(spt_total_bias,1,mean),
                               cv_se=apply(spt_total_bias,1,se),
                               data_name=name)
return(spt_total_bias)
}
```

Minimum sample sizes for CV1 and CV4

```
Min_Sample_Size <- function(data, accuracy) {
    data1 <- data[data$g == 1, ]
    data4 <- data[data$g == 4, ]
    range1 <- data1[data1$cv_bias >= accuracy, ]
    range4 <- data4[data4$cv_bias >= accuracy, ]
    if (length(range1$species) > 0) {
        min_ss1 <- min(range1$sample_size)
    } else {
        min_ss1 <- 400
    }
    min_ss4 <- min(range4$sample_size)
    min_ss <- c(min_ss1, min_ss4)
    names(min_ss) <- c("CV1", "CV4")
    return(min_ss)
}
```

Line chart

```
# figure for sample size and cv_bias
New_Line_chart <- function(cv_result, title, x_lab, y_lab) {

    p <- ggplot(cv_result, aes(x = log(sample_size), y = cv_bias, col = Estimator)) +
      geom_point(size = 0.8, aes(shape = Estimator)) +
      geom_line(aes(linetype = Data_normalization)) +
      geom_errorbar(aes(ymin = cv_bias - cvbias_se, ymax = cv_bias + cvbias_se, col = Estim
ator), width = 0.04) +
      scale_linetype_manual(values = c("dotted", "solid")) +
      ggtitle(title) + xlab(x_lab) + ylab(y_lab)   +
      scale_x_continuous(breaks = c(log(10), log(20), log(40), log(80), log(170), log(400),
 log(1000)),
                         labels = c(10, 20, 40, 80, 170, 400, 1000)) +
      theme(plot.title = element_text(hjust = 0, size = 13),
            axis.title.x = element_text(size = 13),
            axis.title.y = element_text(size = 13),
            legend.position = c(0.89, 0.35),
            legend.text = element_text(size = 9, face = "italic")) +
      theme(panel.border = element_rect(fill = "transparent", color = "black"),
            panel.grid.minor = element_blank(),
            panel.grid.major = element_blank(),
            panel.background = element_rect(fill = "transparent", color = "white"))
    p1 <- p + scale_colour_colorblind()
    #p2 <- p1 + scale_shape_discrete(labels = expression("CV"[1], "CV"[2], "CV"[3], "CV"[4]
, "CV"[5], "CV"[6])) + scale_color_discrete(labels = expression("CV"[1], "CV"[2], "CV"[3],
"CV"[4], "CV"[5], "CV"[6]))

    return(p1)
}


x_lab_l <- "Sample size"
y_lab_l <- "Proportional bias"

x_lab_b <- "Data normalization"
y_lab_b <- "Sum of absolute proportional bias across sample sizes"
```

Bar chart

```
New_Bar_chart <- function(total_bias, title, x_lab, y_lab) {

    b <- ggplot(total_bias, aes(x = Data_normalization, y = total_bias, fill = Estimator))
+
      geom_bar(stat = "identity", position = "dodge", width = 0.6) +
      geom_errorbar(aes(ymin = total_bias - cv_se, ymax = total_bias + cv_se), size = 0.3,
width = 0.1, position = position_dodge(0.6)) +
      ggtitle(title) + xlab(x_lab) + ylab(y_lab) +
      theme(plot.title = element_text(hjust = 0, size = 13),
            axis.title.x = element_text(size = 13),
            axis.title.y = element_text(size = 13),
            legend.position = c(0.4, 0.5),) +
      theme(panel.border = element_rect(fill = "transparent", color = "black"),
            panel.grid.minor = element_blank(),
            panel.grid.major = element_blank(),
            panel.background = element_rect(fill = "transparent", color = "white"))
```

```
     b1 <- b + scale_fill_colorblind()
     #b2 <- b1 + scale_fill_discrete(labels = expression("CV"[1], "CV"[2], "CV"[3], "CV"[4]
, "CV"[5], "CV"[6]))

    return(b1)
}
```

Trait Density chart

```
Density_chart <- function(data, trait, title, x_lab_den, y_lab_den) {

    p <- ggplot(data, mapping = aes(x = data[, colnames(data) == trait], group = sp, col =
factor(sp))) +
      geom_line(stat = "density", size = 0.5)+
      ggtitle(title) + xlab(x_lab_den) + ylab(y_lab_den) +
      theme(plot.title = element_text(hjust = 0, size = 11),
            axis.title.x = element_text(size = 13),
            axis.title.y = element_text(size = 13),
            legend.position = c(0.75, 0.6),
            legend.text = element_text(size = 7.5, face = "italic"),
            legend.title = element_blank(),
            legend.key.size = unit(0.4, "cm"),
            legend.key.width = unit(0.4, "cm")) +
      theme(panel.border = element_rect(fill = "transparent", color = "black"),
            panel.grid.minor = element_blank(), panel.grid.major = element_blank(),
            panel.background = element_rect(fill = "transparent", color = "white"))
    p1 <- p + scale_color_colorblind()

    return(p1)
}
```

Trait boxplot

```
Boxplot <- function(data, trait, title, x_lab_box, y_lab_box) {
    b <- ggplot(data, aes(x = sp, y = data[, colnames(data) == trait], fill = factor(sp)))
+
      geom_boxplot(aes(group = cut_width(factor(sp), 0.25)), notch = TRUE, outlier.size = 3
, coef = 2, outlier.alpha = 0.2) +
      ggtitle(title) + xlab(x_lab_box) + ylab(y_lab_box) +
      theme(legend.position = "none",
            plot.title = element_text(hjust = 0, size = 11),
            axis.title.x = element_text(size = 13),
            axis.title.y = element_text(size = 13),
            axis.text.x = element_text(size = 7, face = "italic", angle = 10),
            axis.text.y = element_text(size = 8)) +
      theme(panel.border = element_rect(fill = "transparent", color = "black"),
            panel.grid.minor = element_blank(), panel.grid.major = element_blank(),
            panel.background = element_rect(fill = "transparent", color = "white"))
    b1 <- b + scale_fill_colorblind()

    return(b1)
}
```

Data Analyses

The simulated trait data

```
if (file.exists("/home/jingyang/labnotes/Work-Log-of-Guochun-Lab/Jing_work_1/sim_results_4d
atasets_rexp1.RData")) {
    load("/home/jingyang/labnotes/Work-Log-of-Guochun-Lab/Jing_work_1/sim_results_4datasets
_rexp1.RData")
    load("/home/jingyang/labnotes/Work-Log-of-Guochun-Lab/Jing_work_1/sim_result_4datasets_
100shapes_5000rep_each7.RData")
} else {

    load("/home/jingyang/labnotes/Work-Log-of-Guochun-Lab/Jing_work_1/Real_Trait_Outliers_3
sd.RData")  # for add outliers similar to real trait datasets
    Nshape <- 100
    Nsample <- seq(10, 1000, by = 10)
    Nrep <- 10000
    Single_Nrep <- 1000
    Cir_Nrep <- Nrep/Single_Nrep  # for calculating se

    # generate simulated trait data incluing shape1 and shape2 for looping computations
    rm(.Random.seed)
    set.seed(11)
    shape_data <- data.frame(Nshape = 1:Nshape, shape1 = NA, shape2 = NA, maxbias = NA)
    trait_all <- matrix(NA, nrow = Nshape, ncol = 10000)
    for (i in 1:Nshape) {
        shape_data[i, "shape1"] <- runif(1, 1, 10)
        shape_data[i, "shape2"] <- runif(1, 5, 30)
        Noutliers <- 480
        trait_alli <- rgamma(9520, shape_data[i, "shape1"], shape_data[i, "shape2"])
        all_maxbias <- rnorm(99999, mean = mean_maxbiasSD, sd = sd_maxbiasSD)
        shape_data[i, "maxbias"] <- sample(all_maxbias[all_maxbias > 3], 1)
        meani <- mean(trait_alli, na.rm = T)
        sdi <- sd(trait_alli, na.rm=T)
        #add_outliers <- runif(shape_data[i, "P_outliers"], min=meani+2*sdi, max=(shape_dat
a[i, "maxbias"] * sdi + meani))
        add_outliers <- rexp(99999, rate = 1)
        add_outliers <- add_outliers[add_outliers>(meani+3*sdi) & add_outliers<((shape_data
[i, "maxbias"] * sdi + meani))]
        add_outliers <- sample(add_outliers, 480,replace = T)
        # sample(seq(meani, (shape_data[i, "maxbias"] * meani), 1e-04), shape_data[i, "P_ou
tliers"], replace = T)
        # add_outliers <- (maxbias+1)*mean(trait_all)
        trait_all[i, ] <- c(trait_alli, add_outliers) + 1
    }

     # Simlated result Data1: Original data
    sim_result <- Sim_All_CVBias(trait_all, Nsample, Single_Nrep, Cir_Nrep, Nshape)
    save(shape_data, trait_all, sim_result, file = "/home/yangjing/labnotes/sim_results7.RD
ata")
    # Data2: log-transform data
    sim_result_log <- Sim_All_CVBias(log(trait_all), Nsample, Single_Nrep, Cir_Nrep, Nshape
)
    save(shape_data, trait_all, sim_result_log, file = "/home/yangjing/labnotes/sim_results
_log7.RData")
    # Data3: cube-transform data
    sim_result_curt <- Sim_All_CVBias(trait_all^(1/3), Nsample, Single_Nrep, Cir_Nrep, Nsha
pe)
```

```
    save(shape_data, trait_all,  sim_result_curt,  file = "/home/yangjing/labnotes/sim_resu
lts_cube7.RData")
    # Data4: min-max normalization data
    trait_all_01 <- trait_all
    for (i in 1:Nshape) {
        trait_all_01[i, ] <- (trait_all_01[i, ] - min(trait_all_01[i, ]))/(max(trait_all_01
[i, ]) - min(trait_all_01[i, ]))
    }
    sim_result_01 <- Sim_All_CVBias(trait_all_01, Nsample, Single_Nrep, Cir_Nrep, Nshape)
    save(shape_data, trait_all, sim_result, sim_result_log, sim_result_curt, sim_result_01,
 file = "/home/yangjing/labnotes/Work-Log-of-Guochun-Lab//home/jingyang/labnotes/Work-Log-o
f-Guochun-Lab/Jing_work_1/sim_results_4datasets_rexp7.RData")

    # Simlated result Data1: Original data
    sim_result <- Sim_All_CVBias(trait_all, Nsample, Single_Nrep, Cir_Nrep, Nshape)
    # Data2: log-transform data
    sim_result_log <- Sim_All_CVBias(log(trait_all), Nsample, Single_Nrep, Cir_Nrep, Nshape
)
    # Data3: cube-transform data
    sim_result_curt <- Sim_All_CVBias(trait_all^(1/3), Nsample, Single_Nrep, Cir_Nrep, Nsha
pe)
    # Data4: min-max normalization data
    trait_all_01 <- trait_all
    for (i in 1:Nshape) {
        trait_all_01[i, ] <- (trait_all_01[i, ] - min(trait_all_01[i, ]))/(max(trait_all_01
[i, ]) - min(trait_all_01[i, ]))
    }
    sim_result_01 <- Sim_All_CVBias(trait_all_01, Nsample, Single_Nrep, Cir_Nrep, Nshape)

    save(shape_data, trait_all, sim_result, sim_result_log, sim_result_curt, sim_result_01,
 file = "/home/jingyang/labnotes/Work-Log-of-Guochun-Lab/Jing_work_1/sim_results_4datasets_
100shapes_5000rep.RData")

    ############ calculate simluted result for each simluted trait dataset
    Sim_CVBias <- function(trait_all, Nsample, Single_Nrep, Cir_Nrep) {
        mean <- mean(trait_all, na.rm = T)
        max_bias <- (max(trait_all, na.rm = T) - mean)/mean
        l.test <- lillie.test(trait_all)
        D <- l.test$statistic
        P_outliers <- length(trait_all[trait_all > (4 * mean)])/length(trait_all)

        cv <- matrix(NA, nrow = 6 * length(Nsample), ncol = Cir_Nrep)
        total_biass <- matrix(NA, nrow = 6, ncol = Cir_Nrep)
        for (i in 1:Cir_Nrep) {

            cv[, i] <- unlist(mclapply(Nsample, function(x)
              apply(replicate(Single_Nrep, Sim_CV(x, trait_all)), 1, mean), mc.cores = 50))
            total_biass[, i] <- apply(abs(matrix(cv[, i], nrow = 6, ncol = length(Nsample))
), 1, sum)
        }
        cv_bias <- data.frame(g = rep(1:6, length(Nsample)),
                              sample_size = rep(Nsample, each = 6),
                              cvbias = apply(cv, 1, mean),
                              cvbias_se = apply(cv, 1, se))
        total_bias <- data.frame(g = 1:6,
                                 total_bias = apply(total_biass, 1, mean),
                                 cv_se = apply(total_biass, 1, se),
                                 D = rep(D, 6),
```

```
                                max_bias = rep(max_bias, 6),
                                P_outliers = rep(P_outliers, 6))
        return(list(cv_bias = cv_bias, total_bias = total_bias))
    }

    sim_result_each <- list()
    for (s in 1:Nshape) {
        print(paste0("[", s, "]", Sys.time()))
        sim_result_each[[s]] <- Sim_CVBias(trait_all[s, ], Nsample, Single_Nrep, Cir_Nrep)
    }

    sim_result_log_each <- list()
    for (s in 1:Nshape) {
        print(paste0("[", s, "]", Sys.time()))
        sim_result_log_each[[s]] <- Sim_CVBias(log(trait_all[s, ]), Nsample, Single_Nrep, C
ir_Nrep)
    }

    sim_result_01_each <- list()
    for (s in 1:Nshape) {
        print(paste0("[", s, "]", Sys.time()))
        traits <- (trait_all[s, ] - min(trait_all[s, ], na.rm = T))/(max(trait_all[s, ], na
.rm = T) - min(trait_all[s, ], na.rm = T))
        sim_result_01_each[[s]] <- Sim_CVBias(traits, Nsample, Single_Nrep, Cir_Nrep)
    }

    sim_result_curt_each <- list()
    for (s in 1:Nshape) {
        print(paste0("[", s, "]", Sys.time()))
        traits <- (trait_all[s, ])^(1/3)
        sim_result_curt_each[[s]] <- Sim_CVBias(traits, Nsample, Single_Nrep, Cir_Nrep)
    }
    save(shape_data, trait_all, sim_result_each, file = "/home/yangjing/labnotes/Work-Log-o
f-Guochun-Lab//home/jingyang/labnotes/Work-Log-of-Guochun-Lab/Jing_work_1/sim_result_4datas
ets_100shapes_5000rep_each7.RData")
}
```

- **Lilliefor tests of simulated trait value distributions – Table S5**

```
sim_illie_test <- data.frame()
for (i in 1:20) {
    a <- lillie.test(trait_all[i, ])
    b <- lillie.test(log(trait_all[i, ]))
    if (a$p.value < 0.001) {
        nonormal_P <- "<0.001"
    } else {nonormal_P <- round(a$p.value, 2)}
    if (b$p.value < 0.001) {
        log_P <- "<0.001"
    } else {log_P <- round(b$p.value, 2)}
    temp <- data.frame(shape = round(shape_data[i, "shape1"], 2),
                       scale = round(shape_data[i, "shape2"], 2), #P_outliers = shape_data[
i, "P_outliers"],
                       maxbias = shape_data[i, "maxbias"],
                       nonormal_D = round(a$statistic, 2),
                       nonormal_P_value = nonormal_P,
                       log_D = round(b$statistic, 2),
                       log_P_value = log_P)
```

```
    sim_illie_test <- rbind(sim_illie_test, temp)
}
table_s5 <- sim_illie_test
write.csv(table_s5, file = "/home/jingyang/labnotes/Work-Log-of-Guochun-Lab/Jing_work_1/Tab
le S5.csv")
```

Tiantong tree data

- **Load data**

```
tiantong_tree_data <- read.csv("/home/jingyang/labnotes/Work-Log-of-Guochun-Lab/Jing_work_1
/Tiantong_tree_data.csv", header = T)
tiantong_species_name <- c("Eurya loquaian", "Litsea elongata", "Camellia fraterna", "Disty
lium myricoides",
                           "Neolitsea aurata", "Adinandra millettii", "Symplocos anomala")
tiantong_trait_name <- c("SLA", "MLA", "LDMC", "Height")
tiantong_Nsample <- seq(10, 400, 5)
tiantong_Nrep <- 9999
Single_Nrep <- 303
Cir_Nrep <- tiantong_Nrep/Single_Nrep
```

- **Calculate CV**

```
# tiantong result based on original trait dataset
if (file.exists(file = "/home/jingyang/labnotes/Work-Log-of-Guochun-Lab/Jing_work_1/tt_resu
lt.RData")) {
    load("/home/jingyang/labnotes/Work-Log-of-Guochun-Lab/Jing_work_1/tt_result.RData")
} else {
    #set seed for repeatable
    set.seed(1)
    # results of cv bias with se for each species and each trait
    tiantong_result <- Trait_CV(tiantong_tree_data, tiantong_species_name, tiantong_trait_n
ame,
                                tiantong_Nsample, tiantong_Nrep, "Tiantong tree data")

    # results of summary cv bias with se of "Cir_Nrep" times
    Single_Nrep <- 303
    Cir_Nrep <- tiantong_Nrep/Single_Nrep
    tt_result99 <- list()
    for(i in 1:Cir_Nrep){
      print(paste(i,Sys.time()))
      tt_result99[[i]] <- Trait_CV(tiantong_tree_data, tiantong_species_name, tiantong_trai
t_name,
                                   tiantong_Nsample, Single_Nrep, "Tiantong tree data")
    }

    tt_all_bias <- All_CVBias(tt_result99, tiantong_Nsample, "Tiantong tree data")
    tt_all_cv_bias <- tt_all_bias[[1]]
    tt_all_total_bias <- tt_all_bias[[2]]

    tt_sp_bias <- Species_CVBias(tt_result99, tiantong_Nsample, tiantong_species_name, "Tia
ntong tree data")
    tt_sp_cv_bias <- tt_sp_bias[[1]]
    tt_sp_total_bias <- tt_sp_bias[[2]]

    tt_trait_bias <- Trait_CVBias(tt_result99, tiantong_Nsample, tiantong_trait_name, "Tian
```

```
tong tree data")
    tt_trait_cv_bias <- tt_trait_bias[[1]]
    tt_trait_total_bias <- tt_trait_bias[[2]]

    tt_spt_total_bias <- Spt_CVBias(tt_result99, tiantong_Nsample, tiantong_species_name,
                                    tiantong_trait_name, "Tiantong tree data")

    save(tiantong_result,tt_result303, tt_result99, tt_all_cv_bias, tt_all_total_bias,
         tt_sp_cv_bias, tt_sp_total_bias, tt_trait_cv_bias, tt_trait_total_bias, tt_spt_tot
al_bias,
         file ="/home/jingyang/labnotes/Work-Log-of-Guochun-Lab/Jing_work_1/tt_result.RData
")

}

# tiantong result based on log trait dataset
if (file.exists(file = "/home/jingyang/labnotes/Work-Log-of-Guochun-Lab/Jing_work_1/tt_log_
result.RData")) {
    load("/home/jingyang/labnotes/Work-Log-of-Guochun-Lab/Jing_work_1/tt_log_result.RData")
} else {
    # Method: data standardization (Log-transform)
    tt_log_data <- tiantong_tree_data
    tt_log_data$SLA <- log(tiantong_tree_data$SLA)
    tt_log_data$MLA <- log(tiantong_tree_data$MLA)
    tt_log_data$LDMC <- log(tiantong_tree_data$LDMC)
    tt_log_data$Height <- log(tiantong_tree_data$Height)
    rm(.Random.seed)
    set.seed(1)
    tt_log_result <- Trait_CV(tt_log_data, tiantong_species_name, tiantong_trait_name,
                              tiantong_Nsample, tiantong_Nrep, "Tiantong tree data")

    tt_log_result303 <- list()
    for (i in 1:Cir_Nrep) {
        tt_log_result303[[i]] <- Trait_CV(tt_log_data, tiantong_species_name, tiantong_trai
t_name,
                                          tiantong_Nsample, Single_Nrep, "Tiantong tree dat
a")
    }
    tt_all_bias <- All_CVBias(tt_log_result303, tiantong_Nsample, "Tiantong tree data")
    tt_log_Acv_bias <- tt_all_bias[[1]]
    tt_log_Atotal_bias <- tt_all_bias[[2]]
    tt_sp_bias <- Species_CVBias(tt_log_result303, tiantong_Nsample, tiantong_species_name,
 "Tiantong tree data")
    tt_log_Scv_bias <- tt_sp_bias[[1]]
    tt_log_Stotal_bias <- tt_sp_bias[[2]]

    tt_trait_bias <- Trait_CVBias(tt_log_result303, tiantong_Nsample, tiantong_trait_name,
"Tiantong tree data")
    tt_log_Tcv_bias <- tt_trait_bias[[1]]
    tt_log_Ttotal_bias <- tt_trait_bias[[2]]

    tt_log_STtotal_bias <- Spt_CVBias(tt_log_result303, tiantong_Nsample, tiantong_species_
name,
                                    tiantong_trait_name, "Tiantong tree data")

    save(tt_log_data, tt_log_result, tt_log_result303, tt_log_Acv_bias, tt_log_Atotal_bias,

         tt_log_Scv_bias, tt_log_Stotal_bias, tt_log_Tcv_bias, tt_log_Ttotal_bias,tt_log_ST
```

```
total_bias,
          file = "/home/jingyang/labnotes/Work-Log-of-Guochun-Lab/Jing_work_1/tt_log_result
.RData")
}


if (file.exists(file = "/home/jingyang/labnotes/Work-Log-of-Guochun-Lab/Jing_work_1/tt_Cube
01_result.RData")) {
    load("/home/jingyang/labnotes/Work-Log-of-Guochun-Lab/Jing_work_1/tt_Cube01_result.RDat
a")
} else {

   # Method2: data standardization (Min-Max normalization)
    tt_normal_data <- tiantong_tree_data
    for (i in 1:length(tiantong_trait_name)) {
        traiti <- tt_normal_data[, colnames(tt_normal_data) == tiantong_trait_name[i]]
        tt_normal_data[, colnames(tt_normal_data) == tiantong_trait_name[i]] <- (traiti - m
in(traiti, na.rm = T))/(max(traiti, na.rm = T) - min(traiti, na.rm = T))
    }  #range(0,1)

    set.seed(1)
    tt_normal_result <- Trait_CV(tt_normal_data, tiantong_species_name, tiantong_trait_name
,
                                 tiantong_Nsample, tiantong_Nrep, "Tiantong tree data")

    tt_normal_result303 <- list()
    for (i in 1:Cir_Nrep) {
        tt_normal_result303[[i]] <- Trait_CV(tt_normal_data, tiantong_species_name, tianton
g_trait_name,
                                             tiantong_Nsample, Single_Nrep, "Tiantong tree
data")
    }
    tt_all_bias <- All_CVBias(tt_normal_result303, tiantong_Nsample, "Tiantong tree data")
    tt_normal_Acv_bias <- tt_all_bias[[1]]
    tt_normal_Atotal_bias <- tt_all_bias[[2]]

    # Method3: data standardization (Cube-root transform)
    tiantong_tree_data <- read.csv("/home/jingyang/labnotes/Work-Log-of-Guochun-Lab/Jing_wo
rk_1/Tiantong_tree_data.csv", header = T)
    tt_cube_data <- tiantong_tree_data
    tt_cube_data$SLA <- (tiantong_tree_data$SLA)^(1/3)
    tt_cube_data$MLA <- (tiantong_tree_data$MLA)^(1/3)
    tt_cube_data$LDMC <- (tiantong_tree_data$LDMC)^(1/3)
    tt_cube_data$Height <- (tiantong_tree_data$Height)^(1/3)

    set.seed(1)
    tt_cube_result <- Trait_CV(tt_cube_data, tiantong_species_name, tiantong_trait_name,
                               tiantong_Nsample, tiantong_Nrep, "Tiantong tree data")

    tt_cube_result303 <- list()
    for (i in 1:Cir_Nrep) {
        tt_cube_result303[[i]] <- Trait_CV(tt_cube_data, tiantong_species_name, tiantong_tr
ait_name,
                                           tiantong_Nsample, Single_Nrep, "Tiantong tree da
ta")
    }
```

```
    tt_all_bias <- All_CVBias(tt_cube_result303, tiantong_Nsample, "Tiantong tree data")
    tt_cube_Acv_bias <- tt_all_bias[[1]]
    tt_cube_Atotal_bias <- tt_all_bias[[2]]

    save(tt_normal_data, tt_cube_data,tt_cube_result, tt_cube_result303, tt_cube_Acv_bias, 
tt_cube_Atotal_bias,
         tt_normal_result, tt_normal_result303, tt_normal_Acv_bias, tt_normal_Atotal_bias,
         file = "/home/jingyang/labnotes/Work-Log-of-Guochun-Lab/Jing_work_1/tt_Cube01_resu
lt.RData")
}
```

- **Calculate minimum sample size – Table 2.**

```
tt_min_sample_size <- data.frame(Species = rep(tiantong_species_name, each=length(tiantong_
trait_name)),
                                 Trait = rep(tiantong_trait_name, length(tiantong_species_n
ame)),
                                 Nonormal_CV1 = as.numeric(NA),
                                 Nonormal_CV4 = as.numeric(NA),
                                 Log_CV1 = as.numeric(NA),
                                 Log_CV4 = as.numeric(NA))

trait_min_nonormal <- c()
trait_min_log <- c()
for (i in 1:length(tiantong_species_name)) {
    spi_nonormal <- tiantong_result[tiantong_result$species == tiantong_species_name[i], ]
    spi_log <- tt_log_result[tt_log_result$species == tiantong_species_name[i], ]

    for (j in 1:length(tiantong_trait_name)) {
        min_nonormal <- Min_Sample_Size(spi_nonormal[spi_nonormal$trait == tiantong_trait_n
ame[j], ], -0.05)
        min_log <- Min_Sample_Size(spi_log[spi_log$trait == tiantong_trait_name[j], ], -0.0
5)
        trait_min_nonormal <- c(trait_min_nonormal, min_nonormal)
        trait_min_log <- c(trait_min_log, min_log)
    }
}
 tt_min_sample_size[,3:4] <- matrix(trait_min_nonormal, nrow = length(tiantong_species_name
) * length(tiantong_trait_name), ncol=2, byrow=T)
 tt_min_sample_size[,5:6] <- matrix(trait_min_log, nrow = length(tiantong_species_name) * l
ength(tiantong_trait_name), ncol=2, byrow=T)
write.csv(tt_min_sample_size, file = "/home/jingyang/labnotes/Work-Log-of-Guochun-Lab/Jing_
work_1/Table 2.csv")
```

- **Lilliefor tests of trait value distributions for four traits of each species – Table S3**

```
tt_lillie_test <- data.frame()
for (i in 1:length(tiantong_species_name)) {
    spi <- tiantong_tree_data[tiantong_tree_data$sp == tiantong_species_name[i], ]
    D <- c()
    P <- c()
    for (j in 1:length(tiantong_trait_name)) {
        temp_test <- lillie.test(spi[, colnames(spi) == tiantong_trait_name[j]])
        D <- c(D, temp_test$statistic)
        if(temp_test$p.value < 0.001){
          p = "<0.001"
        }else{p = round(temp_test$p.value,3)}
```

```
        P <- c(P, p)
        P <- c(P, p)
    }
    temp <- data.frame(Data_normalization = "No",
                       Species = tiantong_species_name[i],
                       SLA_D = D[1], SLA_P_value = P[1],
                       MLA_D = D[2], MLA_P_value = P[2],
                       LDMC_D = D[3], LDMC_P_value = P[3],
                       Height_D = D[4], Height_P_value = P[4])
    tt_lillie_test <- rbind(tt_lillie_test, temp)
}


tt_lillie_test_log <- data.frame()
for (i in 1:length(tiantong_species_name)) {
    spi <- tt_log_data[tt_log_data$sp == tiantong_species_name[i], ]
    D <- c()
    P <- c()
    for (j in 1:length(tiantong_trait_name)) {
        temp_test <- lillie.test(spi[, colnames(spi) == tiantong_trait_name[j]])
        D <- c(D, temp_test$statistic)
        if(temp_test$p.value < 0.001){
          p = "<0.001"
        }else{p = round(temp_test$p.value,3)}
        P <- c(P, p)
    }
    temp <- data.frame(Data_normalization = "Log-transform",
                       Species = tiantong_species_name[i],
                       SLA_D = D[1], SLA_P_value = P[1],
                       MLA_D = D[2], MLA_P_value = P[2],
                       LDMC_D = D[3], LDMC_P_value = P[3],
                       Height_D = D[4], Height_P_value = P[4])
    tt_lillie_test_log <- rbind(tt_lillie_test_log, temp)
}

table_s3 <- rbind(tt_lillie_test, tt_lillie_test_log)
write.csv(tt_lillie_test_log, file = "/home/jingyang/labnotes/Work-Log-of-Guochun-Lab/Jing_
work_1/Table S3.csv")
write.csv(table_s3, file = "/home/jingyang/labnotes/Work-Log-of-Guochun-Lab/Jing_work_1/Tab
le S3_2conditions.csv")
```

- **Study case**
- **find minimum sample size through slope**

Ningbo tree data

- **Load data**

```
ningbo_tree_data <- read.csv("/home/jingyang/labnotes/Work-Log-of-Guochun-Lab/Jing_work_1/N
ingbo_tree_data.csv", header = T)
ningbo_species_name <- as.character(unique(ningbo_tree_data$sp))
ningbo_trait_name <- c("SLA", "LDMC")
ningbo_Nsample <- seq(10, 150, 5)
ningbo_Nrep <- 9999
```

- **Calculate CV**

```
if (file.exists(file = "/home/jingyang/labnotes/Work-Log-of-Guochun-Lab/Jing_work_1/nb_resu
lt.RData")) {
    load("/home/jingyang/labnotes/Work-Log-of-Guochun-Lab/Jing_work_1/nb_result.RData")
} else {
    #set seed for repeatable
    set.seed(1)
    ningbo_result <- Trait_CV(ningbo_tree_data, ningbo_species_name, ningbo_trait_name,
                              ningbo_Nsample, ningbo_Nrep, "Ningbo tree data")

     # results of summary cv bias with se of "Cir_Nrep" times
    Single_Nrep <- 99
    Cir_Nrep <- ningbo_Nrep/Single_Nrep
    nb_result99 <- list()
    for(i in 1:Cir_Nrep){
      print(i)
      nb_result99[[i]] <- Trait_CV(ningbo_tree_data, ningbo_species_name, ningbo_trait_name
,
                                   ningbo_Nsample, Single_Nrep, "Ningbo tree data")
    }

    nb_all_bias <- All_CVBias(nb_result99, ningbo_Nsample, "Ningbo tree data")
    nb_all_cv_bias <- nb_all_bias[[1]]
    nb_all_total_bias <- nb_all_bias[[2]]

    nb_sp_bias <- Species_CVBias(nb_result99, ningbo_Nsample, ningbo_species_name, "Ningbo
tree data")
    nb_sp_cv_bias <- nb_sp_bias[[1]]
    nb_sp_total_bias <- nb_sp_bias[[2]]

    nb_trait_bias <- Trait_CVBias(nb_result99, ningbo_Nsample, ningbo_trait_name, "Ningbo t
ree data")
    nb_trait_cv_bias <- nb_trait_bias[[1]]
    nb_trait_total_bias <- nb_trait_bias[[2]]

    nb_spt_total_bias <- Spt_CVBias(nb_result99, ningbo_Nsample, ningbo_species_name,
                                    ningbo_trait_name, "Ningbo tree data")

    save(ningbo_result, nb_result99, nb_all_cv_bias, nb_all_total_bias, nb_sp_cv_bias,
         nb_sp_total_bias, nb_trait_cv_bias, nb_trait_total_bias, nb_spt_total_bias,
         file ="/home/jingyang/labnotes/Work-Log-of-Guochun-Lab/Jing_work_1/nb_result.RData
")
}

# result based on log-transform dataset
 if (file.exists(file = "/home/jingyang/labnotes/Work-Log-of-Guochun-Lab/Jing_work_1/nb_log
_result.RData")) {
    load("/home/jingyang/labnotes/Work-Log-of-Guochun-Lab/Jing_work_1/nb_log_result.RData")
} else {
    nb_log_data <- ningbo_tree_data
    nb_log_data$SLA <- log(ningbo_tree_data$SLA)
    nb_log_data$LDMC <- log(ningbo_tree_data$LDMC+1)

    nb_log_result <- Trait_CV(nb_log_data, ningbo_species_name, ningbo_trait_name,
                              ningbo_Nsample, ningbo_Nrep, "ningbo tree data")

    nb_log_result303 <- list()
    for (i in 1:Cir_Nrep) {
```

```
      print(paste0(i,Sys.time()))
        nb_log_result303[[i]] <- Trait_CV(nb_log_data, ningbo_species_name, ningbo_trait_na
me,
                                          ningbo_Nsample, Single_Nrep, "ningbo tree data")
    }
    nb_all_bias <- All_CVBias(nb_log_result303, ningbo_Nsample, "ningbo tree data")
    nb_log_Acv_bias <- nb_all_bias[[1]]
    nb_log_Atotal_bias <- nb_all_bias[[2]]

    nb_sp_bias <- Species_CVBias(nb_log_result303, ningbo_Nsample, ningbo_species_name, "Ni
ngbo tree data")
    nb_log_Scv_bias <- nb_sp_bias[[1]]
    nb_log_Stotal_bias <- nb_sp_bias[[2]]

    nb_trait_bias <- Trait_CVBias(nb_log_result303, ningbo_Nsample, ningbo_trait_name, "Nin
gbo tree data")
    nb_log_Tcv_bias <- nb_trait_bias[[1]]
    nb_log_Ttotal_bias <- nb_trait_bias[[2]]

    nb_log_STtotal_bias <- Spt_CVBias(nb_log_result303, ningbo_Nsample, ningbo_species_name
,
                                    ningbo_trait_name, "Ningbo tree data")

    save(nb_log_data, nb_log_result, nb_log_result303, nb_log_Acv_bias, nb_log_Atotal_bias,

         nb_log_Scv_bias, nb_log_Stotal_bias, nb_log_Tcv_bias, nb_log_Ttotal_bias,nb_log_ST
total_bias,
         file = "/home/jingyang/labnotes/Work-Log-of-Guochun-Lab/Jing_work_1/nb_log_result.
RData")
}

if (file.exists(file = "/home/jingyang/labnotes/Work-Log-of-Guochun-Lab/Jing_work_1/nb_Cube
01_result.RData")) {
    load("/home/jingyang/labnotes/Work-Log-of-Guochun-Lab/Jing_work_1/nb_Cube01_result.RDat
a")
} else {
  Single_Nrep <- 303
  Cir_Nrep <- ningbo_Nrep/Single_Nrep
  nb_normal_data <- ningbo_tree_data
  for (i in 1:length(ningbo_trait_name)) {
        traiti <- nb_normal_data[, colnames(nb_normal_data) == ningbo_trait_name[i]]
        nb_normal_data[, colnames(nb_normal_data) == ningbo_trait_name[i]] <- (traiti - min
(traiti, na.rm = T))/(max(traiti, na.rm = T) - min(traiti, na.rm = T))
    }  #range(0,1)

    set.seed(1)
    nb_normal_result <- Trait_CV(nb_normal_data, ningbo_species_name, ningbo_trait_name,
                                 ningbo_Nsample, ningbo_Nrep, "ningbo tree data")

    nb_normal_result303 <- list()
    for (i in 1:Cir_Nrep) {
        nb_normal_result303[[i]] <- Trait_CV(nb_normal_data, ningbo_species_name, ningbo_tr
ait_name,
                                             ningbo_Nsample, Single_Nrep, "ningbo tree data
")
    }
    nb_all_bias <- All_CVBias(nb_normal_result303, ningbo_Nsample, "ningbo tree data")
    nb_normal_Acv_bias <- nb_all_bias[[1]]
```

```
    nb_normal_Atotal_bias <- nb_all_bias[[2]]

    # Method3: data standardization (Cube-root transform)
    #ningbo_tree_data <- read.csv("/home/jingyang/labnotes/Work-Log-of-Guochun-Lab/Jing_wor
k_1/ningbo_tree_data.csv", header = T)
    nb_cube_data <- ningbo_tree_data
    nb_cube_data$SLA <- (ningbo_tree_data$SLA)^(1/3)
    nb_cube_data$LDMC <- (ningbo_tree_data$LDMC)^(1/3)

    set.seed(1)
    nb_cube_result <- Trait_CV(nb_cube_data, ningbo_species_name, ningbo_trait_name,
                               ningbo_Nsample, ningbo_Nrep, "ningbo tree data")

    nb_cube_result303 <- list()
    for (i in 1:Cir_Nrep) {
        nb_cube_result303[[i]] <- Trait_CV(nb_cube_data, ningbo_species_name, ningbo_trait_
name,
                                           ningbo_Nsample, Single_Nrep, "ningbo tree data")
    }

    nb_all_bias <- All_CVBias(nb_cube_result303, ningbo_Nsample, "ningbo tree data")
    nb_cube_Acv_bias <- nb_all_bias[[1]]
    nb_cube_Atotal_bias <- nb_all_bias[[2]]

    save(nb_normal_data, nb_cube_data,nb_cube_result, nb_cube_result303, nb_cube_Acv_bias,
nb_cube_Atotal_bias,
         nb_normal_result, nb_normal_result303, nb_normal_Acv_bias, nb_normal_Atotal_bias,
         file = "/home/jingyang/labnotes/Work-Log-of-Guochun-Lab/Jing_work_1/nb_Cube01_resu
lt.RData")
}
```

- **Calculate minimum sample size – table S2.**

```
trait_min <- c()
trait_min_log <- c()
for (i in 1:length(ningbo_trait_name)) {
    spi <- ningbo_result[ningbo_result$trait == ningbo_trait_name[i], ]
    spi_log <- nb_log_result[nb_log_result$trait == ningbo_trait_name[i], ]

    for (j in 1:length(ningbo_species_name)) {
        min1 <- Min_Sample_Size(spi[spi$species == ningbo_species_name[j], ], -0.05)
        min1_log <- Min_Sample_Size(spi_log[spi_log$species == ningbo_species_name[j], ], -
0.05)

        trait_min <- c(trait_min, min1)
        trait_min_log <- c(trait_min_log, min1_log)
    }

}
nb_min_sample_size <- data.frame(Bias = rep("±5%", each = length(ningbo_species_name) *
                                              length(ningbo_trait_name)),
                                 Type = "Tree",
                                 Species = rep(ningbo_species_name, length(ningbo_trait_nam
e)),
                                 Trait = rep(ningbo_trait_name, each = length(ningbo_specie
s_name)),
                                 Nonormal_CV1 = as.numeric(trait_min[names(trait_min)=="CV1
```

```
"]),
                                 Nonormal_CV4 = as.numeric(trait_min[names(trait_min)=="CV4
"]),
                                 Log_CV1 = as.numeric(trait_min_log[names(trait_min_log)=="
CV1"]),
                                 Log_CV4 = as.numeric(trait_min_log[names(trait_min_log)=="
CV4"]))
```

- **Lilliefor tests of trait value distributions for two traits of each species –Table S4.**

```
D <- c()
P <- c()
for (i in 1:length(ningbo_trait_name)) {
    for (j in 1:length(ningbo_species_name)) {
        temp_test <- lillie.test(ningbo_tree_data[ningbo_tree_data$sp == ningbo_species_nam
e[j],
                                                  colnames(ningbo_tree_data) == ningbo_trai
t_name[i]])
        D <- c(D, temp_test$statistic)
        if(temp_test$p.value < 0.001){
          p = "<0.001"
        }else{p = round(temp_test$p.value,3)}
        P <- c(P, p)
    }
}


log_D <- c()
log_P <- c()
for (i in 1:length(ningbo_trait_name)) {
    for (j in 1:length(ningbo_species_name)) {
        temp_test <- lillie.test(nb_log_data[nb_log_data$sp == ningbo_species_name[j],
                                                  colnames(nb_log_data) == ningbo_trait_nam
e[i]])
        log_D <- c(log_D, temp_test$statistic)
      if(temp_test$p.value < 0.001){
          p = "<0.001"
        }else{p = round(temp_test$p.value,3)}
        log_P <- c(log_P, p)

    }
}

nb_lillie_test <- data.frame(Type = "Tree",
                             Species = rep(ningbo_species_name, length(ningbo_trait_name)),

                             Trait = rep(ningbo_trait_name, each = length(ningbo_species_na
me)),
                             Nonormal_D = round(D,2), Nonormal_P_value = P,
                               Log_D = round(log_D,2), log_P_value = log_P)
```

Mountain frog data

- **Load data**

```
mountain_frog_data <- read.csv("/home/jingyang/labnotes/Work-Log-of-Guochun-Lab/Jing_work_1
```

```
/Mountain_frog_data.csv", header = T)
frog_species_name <- as.character(unique(mountain_frog_data$sp))
frog_trait_name <- c("HL", "IOD", "TYD", "OPTW", "TW")
frog_Nsample <- seq(10, 400, 5)
frog_Nrep <- 9999
```

- **Calculate CV**

```
if (file.exists(file = "/home/jingyang/labnotes/Work-Log-of-Guochun-Lab/Jing_work_1/frog_re
sult.RData")) {
    load("/home/jingyang/labnotes/Work-Log-of-Guochun-Lab/Jing_work_1/frog_result.RData")
} else {
    #set seed for repeatable
    set.seed(1)
    frog_result <- Trait_CV(mountain_frog_data, frog_species_name, frog_trait_name,
                            frog_Nsample, frog_Nrep, "Mountain frog data")

      # results of summary cv bias with se of "Cir_Nrep" times
    Single_Nrep <- 99
    Cir_Nrep <- frog_Nrep/Single_Nrep
    frog_result99 <- list()
    for(i in 1:Cir_Nrep){
      print(i)
      frog_result99[[i]] <- Trait_CV(mountain_frog_data, frog_species_name, frog_trait_name
,
                                    frog_Nsample, Single_Nrep, "Mountain frog data")
    }

    frog_all_bias <- All_CVBias(frog_result99, frog_Nsample, "Mountain frog data")
    frog_all_cv_bias <- frog_all_bias[[1]]
    frog_all_total_bias <- frog_all_bias[[2]]

    frog_sp_bias <- Species_CVBias(frog_result99, frog_Nsample, frog_species_name, "Mountai
n frog data")
    frog_sp_cv_bias <- frog_sp_bias[[1]]
    frog_sp_total_bias <- frog_sp_bias[[2]]

    frog_trait_bias <- Trait_CVBias(frog_result99, frog_Nsample, frog_trait_name, "Mountain
 frog data")
    frog_trait_cv_bias <- frog_trait_bias[[1]]
    frog_trait_total_bias <- frog_trait_bias[[2]]

    frog_spt_total_bias <- Spt_CVBias(frog_result99, frog_Nsample, frog_species_name,
                                    frog_trait_name, "Mountain frog data")

    save(frog_result, frog_result99, frog_all_cv_bias, frog_all_total_bias, frog_sp_cv_bias
,
         frog_sp_total_bias, frog_trait_cv_bias, frog_trait_total_bias,
         file ="/home/jingyang/labnotes/Work-Log-of-Guochun-Lab/Jing_work_1/frog_result.RDa
ta")
}

# frog result based on log-transform frog trait dataset
if (file.exists(file = "/home/jingyang/labnotes/Work-Log-of-Guochun-Lab/Jing_work_1/frog_lo
g_result.RData")) {
    load("/home/jingyang/labnotes/Work-Log-of-Guochun-Lab/Jing_work_1/frog_log_result.RData
")
```

```
} else {
#### Frog data
    frog_log_data <- mountain_frog_data
    frog_log_data$HL <- log(mountain_frog_data$HL)
    frog_log_data$IOD <- log(mountain_frog_data$IOD)
    frog_log_data$TYD <- log(mountain_frog_data$TYD)
    frog_log_data$OPTW <- log(mountain_frog_data$OPTW)
    frog_log_data$TW <- log(mountain_frog_data$TW)

    frog_log_result <- Trait_CV(frog_log_data, frog_species_name, frog_trait_name,
                          frog_Nsample, frog_Nrep, "frog tree data")

    frog_log_result303 <- list()
    for (i in 1:Cir_Nrep) {
      print(paste0(i,Sys.time()))
        frog_log_result303[[i]] <- Trait_CV(frog_log_data, frog_species_name, frog_trait_na
me,
                                          frog_Nsample, Single_Nrep, "frog tree data")
    }
    frog_all_bias <- All_CVBias(frog_log_result303, frog_Nsample, "frog tree data")
    frog_log_Acv_bias <- frog_all_bias[[1]]
    frog_log_Atotal_bias <- frog_all_bias[[2]]
    frog_sp_bias <- Species_CVBias(frog_log_result303, frog_Nsample, frog_species_name, "Mo
untain frog data")
    frog_log_Scv_bias <- frog_sp_bias[[1]]
    frog_log_Stotal_bias <- frog_sp_bias[[2]]

    frog_trait_bias <- Trait_CVBias(frog_log_result303, frog_Nsample, frog_trait_name, "Mou
ntain frog data")
    frog_log_Tcv_bias <- frog_trait_bias[[1]]
    frog_log_Ttotal_bias <- frog_trait_bias[[2]]

    frog_log_STtotal_bias <- Spt_CVBias(frog_log_result303, frog_Nsample, frog_species_name
,
                                    frog_trait_name, "Mountain frog data")

    save(frog_log_data, frog_log_result, frog_log_result303, frog_log_Acv_bias, frog_log_At
otal_bias,
         frog_log_Scv_bias, frog_log_Stotal_bias, frog_log_Tcv_bias, frog_log_Ttotal_bias,f
rog_log_STtotal_bias,
          file = "/home/jingyang/labnotes/Work-Log-of-Guochun-Lab/Jing_work_1/frog_log_resu
lt.RData")

}

if (file.exists(file = "/home/jingyang/labnotes/Work-Log-of-Guochun-Lab/Jing_work_1/frog_Cu
be01_result.RData")) {
    load("/home/jingyang/labnotes/Work-Log-of-Guochun-Lab/Jing_work_1/frog_Cube01_result.RD
ata")
} else {
  Single_Nrep <- 303
  Cir_Nrep <- frog_Nrep/Single_Nrep
  frog_normal_data <- mountain_frog_data
  for (i in 1:length(frog_trait_name)) {
        traiti <- frog_normal_data[, colnames(frog_normal_data) == frog_trait_name[i]]
        frog_normal_data[, colnames(frog_normal_data) == frog_trait_name[i]] <- (traiti - m
in(traiti, na.rm = T))/(max(traiti, na.rm = T) - min(traiti, na.rm = T))
    }  #range(0,1)
```

```
    set.seed(1)
    frog_normal_result <- Trait_CV(frog_normal_data, frog_species_name, frog_trait_name,
                             frog_Nsample, frog_Nrep, "Mountain frog data")

    frog_normal_result303 <- list()
    for (i in 1:Cir_Nrep) {
        frog_normal_result303[[i]] <- Trait_CV(frog_normal_data, frog_species_name, frog_tr
ait_name,
                                             frog_Nsample, Single_Nrep, "Mountain frog data
")
    }
    frog_all_bias <- All_CVBias(frog_normal_result303, frog_Nsample, "Mountain frog data")
    frog_normal_Acv_bias <- frog_all_bias[[1]]
    frog_normal_Atotal_bias <- frog_all_bias[[2]]

    # Method3: data standardization (Cube-root transform)
    #frog_tree_data <- read.csv("/home/jingyang/labnotes/Work-Log-of-Guochun-Lab/Jing_work_
1/frog_tree_data.csv", header = T)
    frog_cube_data <- mountain_frog_data
    frog_cube_data$HL <- (mountain_frog_data$HL)^(1/3)
    frog_cube_data$IOD <- (mountain_frog_data$IOD)^(1/3)
    frog_cube_data$TYD <- (mountain_frog_data$TYD)^(1/3)
    frog_cube_data$OPTW <- (mountain_frog_data$OPTW)^(1/3)
    frog_cube_data$TW <- (mountain_frog_data$TW)^(1/3)

    set.seed(1)
    frog_cube_result <- Trait_CV(frog_cube_data, frog_species_name, frog_trait_name,
                           frog_Nsample, frog_Nrep, "Mountain frog data")

    frog_cube_result303 <- list()
    for (i in 1:Cir_Nrep) {
        frog_cube_result303[[i]] <- Trait_CV(frog_cube_data, frog_species_name, frog_trait_
name,
                                           frog_Nsample, Single_Nrep, "Mountain frog data")
    }

    frog_all_bias <- All_CVBias(frog_cube_result303, frog_Nsample, "Mountain frog data")
    frog_cube_Acv_bias <- frog_all_bias[[1]]
    frog_cube_Atotal_bias <- frog_all_bias[[2]]

    save(frog_normal_data, frog_cube_data,frog_cube_result, frog_cube_result303, frog_cube_
Acv_bias, frog_cube_Atotal_bias,frog_normal_result, frog_normal_result303, frog_normal_Acv_
bias, frog_normal_Atotal_bias,
         file = "/home/jingyang/labnotes/Work-Log-of-Guochun-Lab/Jing_work_1/frog_Cube01_re
sult.RData")
}
```

- **Calculate minimum sample size – table S2.**

```
trait_min <- c()
trait_min_log <- c()
for (i in 1:length(frog_trait_name)) {
    min1 <- Min_Sample_Size(frog_result[frog_result$trait == frog_trait_name[i], ], -0.05)
    min1_log <- Min_Sample_Size(frog_log_result[frog_log_result$trait == frog_trait_name[i]
, ], -0.05)
```

```
   trait_min <- c(trait_min, min1)
   trait_min_log <- c(trait_min_log, min1_log)
}

frog_min_sample_size <- data.frame(Bias = rep("±5%", each = length(frog_species_name) * len
gth(frog_trait_name)),
                                   Type = "Frog",
                                   Species = rep(frog_species_name, length(frog_trait_name)
),
                                   Trait = rep(frog_trait_name, each = length(frog_species_
name)),
                                   Nonormal_CV1 = as.numeric(trait_min[names(trait_min)=="C
V1"]),
                                   Nonormal_CV4 = as.numeric(trait_min[names(trait_min)=="C
V4"]),
                                   Log_CV1 = as.numeric(trait_min_log[names(trait_min_log)=
="CV1"]),
                                   Log_CV4 = as.numeric(trait_min_log[names(trait_min_log)=
="CV4"]))

table_s2 <- rbind(nb_min_sample_size, frog_min_sample_size)
write.csv(table_s2, file = "/home/jingyang/labnotes/Work-Log-of-Guochun-Lab/Jing_work_1/Tab
le S2.csv")
```

- **Lilliefor tests of trait value distributions for two traits of each species –Table S4.**

```
D <- c()
P <- c()
for (i in 1:length(frog_trait_name)) {
    for (j in 1:length(frog_species_name)) {
        temp_test <- lillie.test(mountain_frog_data[mountain_frog_data$sp == frog_species_n
ame[j],
                                                    colnames(mountain_frog_data) == frog_tr
ait_name[i]])
        D <- c(D, temp_test$statistic)
         if(temp_test$p.value < 0.001){
          p = "<0.001"
        }else{p = round(temp_test$p.value,3)}
        P <- c(P, p)
    }
}

log_D <- c()
log_P <- c()
for (i in 1:length(frog_trait_name)) {
    for (j in 1:length(frog_species_name)) {
        temp_test <- lillie.test(frog_log_data[frog_log_data$sp == frog_species_name[j],
                                                    colnames(frog_log_data) == frog_trait_n
ame[i]])
        log_D <- c(log_D, temp_test$statistic)
        if(temp_test$p.value < 0.001){
          p = "<0.001"
        }else{p = round(temp_test$p.value,3)}
        log_P <- c(log_P, p)
    }
}
frog_lillie_test <- data.frame(Type = "Frog",
```

```
                                     Species = rep(frog_species_name, length(frog_trait_name)),
                                     Trait = rep(frog_trait_name, each = length(frog_species_name
)),
                                     Nonormal_D = round(D,2), Nonormal_P_value = P,
                                     Log_D = round(log_D,2), log_P_value = log_P)


table_s4 <- rbind(nb_lillie_test, frog_lillie_test)
write.csv(table_s4, file = "/home/jingyang/labnotes/Work-Log-of-Guochun-Lab/Jing_work_1/Tab
le S4.csv")
```

Results

Tables

Table 1.

Table 2.

Minimum sample sizes of the most commonly used ITV estimator ITV1 and our best performed estimator ITV5 for each trait (SLA: specific leaf area, MLA: mean leaf area, LDMC: leaf dry mass content, Height: individual height) of each species with abundance ≥ 400 in the Tiantong tree data.

Table S2.

Minimum sample sizes of the commonly used ITV estimator CV1 and our best estimator CV5 for each trait (SLA: specific leaf area, LDMC: leaf dry mass content, HL: head length, IOD: interorbital distance, TYD: tympanum diameter, OPTW: outer metacarpal tubercle width, TW: tibia width) of four tree species and a frog species. Number of measurements for each trait were ≥ 150 for these four tree species in the Ningbo tree data and ≥ 500 for the Mountain frog data.

Table S3.

Lilliefor tests of trait value distributions for four traits, mean leaf area (MLA), specific leaf area (SLA), leaf dry mass content (LDMC) and individual height (Height) of each species with abundance ≥ 400 in the Tiantong tree data. Numbers in D columns are the corresponding values of D statistic in the Lilliefor test.

Figures

Figure 1

Proportional bias on sample size k (k ∈ {10,20,30,…1000}; k ∈ {10,20,…400}), for clarity only points with regular spacing at ln scale showed here, left column) and sum of absolute proportional bias across all sample size for the ith (i ∈ {1,2,3,4,5,6}) CV estimator based on simulated trait values(a, b) and observed trait values (right column) in Tiantong tree data(c, d). All simulated trait values were randomly drawn from a gamma distribution with parameters β1 and β2, which were two independent random variables following a uniform distribution from 1 to 10 and from 5 to 30, respectively.

```
sim_result[[1]]$Data_normalization <- "No normalization"
sim_result_log[[1]]$Data_normalization <- "Log-transformation"
sim_result2 <- rbind(sim_result[[1]], sim_result_log[[1]])
sim_result2$Data_normalization <- factor(sim_result2$Data_normalization, levels=c("No norma
lization", "Log-transformation"))
colnames(sim_result2)[1] <- "Estimator"
sim_result2$Estimator <- as.factor(sim_result2$Estimator)

sim_result[[2]]$Data_normalization <- "No normalization"
```

```
sim_result_log[[2]]$Data_normalization <- "Log-transformation"
sim_result_curt[[2]]$Data_normalization <- "Cube-root transformation"
sim_result_01[[2]]$Data_normalization <- "Min-max transformation"
sim_total_result2 <- rbind(sim_result[[2]], sim_result_log[[2]], sim_result_curt[[2]], sim_
result_01[[2]])
sim_total_result2$Data_normalization <- factor(sim_total_result2$Data_normalization, levels
=c("No normalization", "Log-transformation", "Min-max transformation", "Cube-root transform
ation"))
colnames(sim_total_result2)[1] <- "Estimator"
sim_total_result2$Estimator <- as.factor(sim_total_result2$Estimator)

simp <- New_Line_chart(sim_result2, "", x_lab_l, y_lab_l) +
  theme(legend.position = c(0.8, 0.35)) +
  annotate("rect", xmin = log(10), xmax = log(1000), ymin = -0.05, ymax = 0,alpha = 0.2)
simb <- New_Bar_chart(sim_total_result2, "", x_lab_b, y_lab_b) + theme(legend.position = c(
0.9,0.8))

pdf(file = "/home/jingyang/labnotes/Work-Log-of-Guochun-Lab/Jing_work_1/Figure 1.pdf", widt
h = 12, height = 6)
ggplot2.multiplot(simp, simb,  cols = 2)
dev.off()
```

Figure 2

```
# The figures of Tiantong tree data
tt_all_cv_bias$Data_normalization <- "No normalization"
tt_log_Acv_bias$Data_normalization <- "Log-transformation"
tt_all_cv_bias2 <- rbind(tt_all_cv_bias, tt_log_Acv_bias)
tt_all_cv_bias2$Data_normalization <- factor(tt_all_cv_bias2$Data_normalization, levels=c("
No normalization", "Log-transformation"))
colnames(tt_all_cv_bias2)[1] <- "Estimator"
tt_all_cv_bias2$Estimator <- as.factor(tt_all_cv_bias2$Estimator)

tt_all_total_bias$Data_normalization <- "No normalization"
tt_log_Atotal_bias$Data_normalization <- "Log-transformation"
tt_cube_Atotal_bias$Data_normalization <- "Cube-root transformation"
tt_normal_Atotal_bias$Data_normalization <- "Min-max transformation"
tt_all_total_bias4 <- rbind(tt_all_total_bias, tt_log_Atotal_bias, tt_cube_Atotal_bias, tt_
normal_Atotal_bias)
tt_all_total_bias4$Data_normalization <- factor(tt_all_total_bias4$Data_normalization, leve
ls=c("No normalization", "Log-transformation", "Min-max transformation", "Cube-root transfo
rmation"))
colnames(tt_all_total_bias4)[1] <- "Estimator"
tt_all_total_bias4$Estimator <- as.factor(tt_all_total_bias4$Estimator)

ttp <- New_Line_chart(tt_all_cv_bias2, "(A) Tiantong tree data", x_lab_l, y_lab_l) +
  theme(legend.position = c(0.8, 0.4)) +
  annotate("rect", xmin = log(10), xmax = log(400), ymin = -0.05, ymax = 0,alpha = 0.2)
ttb <- New_Bar_chart(tt_all_total_bias4, "(B) Tiantong tree data", x_lab_b, y_lab_b) + them
e(legend.position = c(0.88, 0.75))

# Ningbo tree data
nb_all_cv_bias$Data_normalization <- "No normalization"
nb_log_Acv_bias$Data_normalization <- "Log-transformation"
nb_all_cv_bias2 <- rbind(nb_all_cv_bias, nb_log_Acv_bias)
nb_all_cv_bias2$Data_normalization <- factor(nb_all_cv_bias2$Data_normalization, levels=c("
No normalization", "Log-transformation"))
```

```
colnames(nb_all_cv_bias2)[1] <- "Estimator"
nb_all_cv_bias2$Estimator <- as.factor(nb_all_cv_bias2$Estimator)

nb_all_total_bias$Data_normalization <- "No normalization"
nb_log_Atotal_bias$Data_normalization <- "Log-transformation"
nb_cube_Atotal_bias$Data_normalization <- "Cube-root transformation"
nb_normal_Atotal_bias$Data_normalization <- "Min-max transformation"
nb_all_total_bias4 <- rbind(nb_all_total_bias, nb_log_Atotal_bias, nb_cube_Atotal_bias, nb_
normal_Atotal_bias)
nb_all_total_bias4$Data_normalization <- factor(nb_all_total_bias4$Data_normalization, leve
ls=c("No normalization", "Log-transformation", "Min-max transformation", "Cube-root transfo
rmation"))
colnames(nb_all_total_bias4)[1] <- "Estimator"
nb_all_total_bias4$Estimator <- as.factor(nb_all_total_bias4$Estimator)

nbp <- New_Line_chart(nb_all_cv_bias2, "(C) Ningbo tree data", x_lab_l, y_lab_l) + theme(le
gend.position = "none") +
  annotate("rect", xmin = log(10), xmax = log(170), ymin = -0.05, ymax = 0,alpha = 0.2)
nbb <- New_Bar_chart(nb_all_total_bias4, "(D) Ningbo tree data", x_lab_b, y_lab_b) + theme(
legend.position = "none")

 # The figures of mountain frog data
frog_all_cv_bias$Data_normalization <- "No normalization"
frog_log_Acv_bias$Data_normalization <- "Log-transformation"
frog_all_cv_bias2 <- rbind(frog_all_cv_bias, frog_log_Acv_bias)
frog_all_cv_bias2$Data_normalization <- factor(frog_all_cv_bias2$Data_normalization, levels
=c("No normalization", "Log-transformation"))
colnames(frog_all_cv_bias2)[1] <- "Estimator"
frog_all_cv_bias2$Estimator <- as.factor(frog_all_cv_bias2$Estimator)

frog_all_total_bias$Data_normalization <- "No normalization"
frog_log_Atotal_bias$Data_normalization <- "Log-transformation"
frog_cube_Atotal_bias$Data_normalization <- "Cube-root transformation"
frog_normal_Atotal_bias$Data_normalization <- "Min-max transformation"
frog_all_total_bias4 <- rbind(frog_all_total_bias, frog_log_Atotal_bias, frog_cube_Atotal_b
ias, frog_normal_Atotal_bias)
frog_all_total_bias4$Data_normalization <- factor(frog_all_total_bias4$Data_normalization,
levels=c("No normalization", "Log-transformation", "Min-max transformation", "Cube-root tra
nsformation"))
colnames(frog_all_total_bias4)[1] <- "Estimator"
frog_all_total_bias4$Estimator <- as.factor(frog_all_total_bias4$Estimator)

frogp <- New_Line_chart(frog_all_cv_bias2, "(E) Mountain frog data", x_lab_l, y_lab_l) + th
eme(legend.position = "none") +
  annotate("rect", xmin = log(10), xmax = log(400), ymin = -0.05, ymax = 0,alpha = 0.2)
frogb <- New_Bar_chart(frog_all_total_bias4, "(F) Mountain frog data", x_lab_b, y_lab_b) +
theme(legend.position = "none")

pdf(file = "/home/jingyang/labnotes/Work-Log-of-Guochun-Lab/Jing_work_1/Figure 2.pdf", widt
h = 9, height = 12)
ggplot2.multiplot(ttp, ttb, nbp, nbb, frogp, frogb, cols = 2)
dev.off()
ggplot2.multiplot(ttp, ttb, nbp, nbb, frogp, frogb, cols = 2)
```

Figure 3.

Mean proportion of bias of the CV1 (left panels) and our best estimator CV5 (right panels) on sample size k (k ∈ {10,20, …400}) for each functional trait (SLA: specific leaf area, MLA: mean leaf area, LDMC: leaf dry mass content, Height:

individual height) of each species (colored line) with abundance ≥ 400 in the Tiantong tree data. Grey area is the region in which the absolute mean proportion of bias is less than 0.05.

```
# subset results of cv1 and cv4
nonormal_data <- tiantong_result[tiantong_result$g %in%c(1,4), ]
nonormal_data$Data_normalization <- "No normalization"
log_data <- tt_log_result[tt_log_result$g %in% c(1, 4), ]
log_data$Data_normalization <- "Log-transformation"
figs3_data <- rbind(nonormal_data, log_data)

colnames(figs3_data)[2] <- "Trait"
colnames(figs3_data)[4] <- "Estimator"
figs3_data <- figs3_data[figs3_data$Trait == "SLA",]
figs3_data$Data_normalization <- factor(figs3_data$Data_normalization, order = TRUE, levels
 = c("No normalization", "Log-transformation"))
figs3_data[figs3_data$Estimator==1, ]$Estimator = "CV1"
figs3_data[figs3_data$Estimator==4, ]$Estimator = "CV4"

p <- ggplot(figs3_data, aes(log(sample_size), cv_bias, col = species)) + geom_point(size =
0.8, shape = 20) +
  geom_line(aes(linetype=Data_normalization)) + geom_errorbar(aes(ymin=cv_bias-cv_se, ymax=
cv_bias+cv_se), width=.04) +
  scale_linetype_manual(values=c("dotted", "solid")) +
  facet_grid(Trait ~ Estimator, scales = "free", space = "free", labeller = label_both) +
  xlab("Sample Size") + ylab("Proportional bias") +
  theme(axis.title.x = element_text(size = 13), axis.title.y = element_text(size = 13),
        legend.text = element_text(size=10, face = "italic"), legend.position = c(0.88, 0.4
2),
        legend.key.size = unit(0.4, "cm"), legend.key.width = unit(0.4, "cm")) +
  scale_x_continuous(breaks = c(log(10), log(20), log(40), log(80), log(170), log(400)), la
bels = c(10, 20, 40, 80, 170, 400)) +
  theme(panel.border = element_rect(fill = "transparent", color = "black"),
        panel.grid.minor = element_blank(), panel.grid.major = element_blank(),
        panel.background = element_rect(fill = "transparent", color = "white")) +
  annotate("rect", xmin = log(10), xmax = log(400), ymin = -0.05, ymax = 0,alpha = 0.2)+ sc
ale_colour_colorblind()

pdf(file = "/home/jingyang/labnotes/Work-Log-of-Guochun-Lab/Jing_work_1/Figure 3.pdf", heig
ht = 4, width = 8)
p
dev.off()
p
```
